# Early Inoculation of a Multi-Species Probiotic in Piglets–Impacts on the Gut Microbiome and Immune Responses

**DOI:** 10.3390/microorganisms13061292

**Published:** 2025-05-31

**Authors:** Lea Hübertz Birch Hansen, Charlotte Lauridsen, Bea Nielsen, Lisbeth Jørgensen, Anna Schönherz, Nuria Canibe

**Affiliations:** 1 Animal Biosolutions, Novonesis, Biologiens vej 2, 2800 Lyngby, Denmark; 2 Department of Animal Science, Aarhus University, Blichers Allé 20, 8830 Tjele, Denmark; 3 Research and Development, Novonesis, Biologiens vej 2, 2800 Lyngby, Denmark

**Keywords:** probiotic, gut microbiota, newborn piglets, diarrhea rate, immunity

## Abstract

Intestinal diseases in nursery pigs harm health and performance and drive antimicrobial resistance. This study evaluated whether early probiotic inoculation helps piglets to cope with weaning-related gut challenges. The probiotic, containing *Lacticaseibacillus rhamnosus*, *Enterococcus lactis*, *Bifidobacterium longum* subsp. *infantis*, and *Bifidobacterium breve*, was given orally to newborn piglets daily until day 4 and then every other day until weaning at day 28 (at 4 × 10^9^ CFU/dose). The control piglets received a placebo. The results showed that the probiotic pigs had reduced fecal alpha-diversity on day 7 but greater Shannon diversity on day 28 (feces) and day 23 (intestinal contents) compared to those of the control pigs. Beta-diversity analysis showed microbial differences between the groups on day 35. Most zOTUs (zero-radius operational taxonomic units) found to significantly differentiate the two treatment groups were found pre weaning. *Bifidobacterium breve*, *Ligilactobacillus salivarius*, as well as *Clostridium ramosum* were significantly more abundant in the feces of the probiotic pigs more than once. The probiotic pigs had higher expression levels of mucin 2 (MUC2); solute carrier family 5, member 8 (SLC5A8); and interleukin 8 (IL-8) post weaning. In the early post-weaning period, the probiotic pigs had less diarrhea as well as lower cadaverine levels in digesta than the control pigs. In conclusion, early probiotic inoculation may induce lasting immunomodulation via microbial antigen changes, enhancing resilience during challenges, like weaning. Notably, the effects persisted beyond weaning and probiotic cessation.

## 1. Introduction

The first weeks of a pig’s life are very critical. Especially, the weaning transition is considered to be a process having detrimental impacts on the performance and the general state of the health of pigs [1]. Antimicrobial substances are used to a high extent when trying to combat enteric infections in newly weaned piglets [2]. Considering the rising rate of antimicrobial resistance, it is crucial to identify new ways for increasing piglets’ robustness for better coping with the weaning transition. In conventional pig husbandries, piglets are weaned at 3–4 weeks of age. At this age, neither the immune system nor the metabolic activity of young pigs is fully competent [3], making it difficult for pigs to cope with stressors involved in the weaning transition. It is well recognized that weaning impacts the composition of the gut’s microbiome, by which the abundance of *Bacteroidaceae* decreases and the abundances of *Veillonellaceae* and *Prevotellaceae* increase [4,5,6]. Interestingly, Dou et al. [7] established that the composition of the gut’s microbiota during nursing can be a predictor of whether a pig develops post-weaning diarrhea or not.

Both animal and human studies have shown that the establishment of the gut’s microbial community exerts profound effects on future life [8,9,10]. According to studies on germ-free mice, commensal bacteria directly influence the maturation of the immune system [11]. Research suggests that the neonatal period may be considered as a window of opportunity, where programming events supporting the health-compatible establishment of the microbiome may be possible [8,12,13,14]. It is still uncertain whether such early interventions should be focused on changing the composition of the gut’s microbiome in a beneficial way or on introducing microbes capable of beneficially affecting the metabolic phenotype of the host or accelerating the development of the immune system.

Probiotics have been suggested as a promising interventive strategy during the time of the physiological plasticity, aiming toward the eubiotic shaping of the gut’s microbiota and improved immunological responses [15]. Probiotics are defined as “live microorganisms, which, when administered in adequate amounts, confer a health benefit on the host” [16]. Studies have established that administering probiotics in the neonatal period of piglets can enhance intestinal morphology, reduce incidences of diarrhea, and improve growth performance [17,18,19,20,21,22,23,24]. Studies evaluating the effect of an early probiotic intervention on a piglet’s ability to overcome challenges related to the weaning transition have, to some extent, found beneficial effects of early probiotic administration post weaning and after the cessation of the administration. However, the specific mechanism of action is not clear.

To the best of our knowledge, no studies have evaluated the impacts of administering a multi-strain probiotic product within the first hours after birth, and during the initial suckling, on the gut’s microbiotal composition and immune responses in pigs across the pre- and post-weaning periods. Although individual probiotic strains have been shown to confer specific health benefits on piglets, multi-strain combinations offer the potential to combine complementary modes of action, thereby enhancing their overall efficacy. This study investigated the effects of a multi-species probiotic product—comprising strains specifically selected for early inoculation of newborn piglets based on their beneficial and compatible attributes [25]—on gut microbiome composition and immune responses.

The objective was to determine the effects of the early-life administration of this probiotic combination on the composition and function of the gut’s microbiota. We hypothesized that the early inoculation of probiotics in newborn piglets would beneficially influence the gut’s microbiotal development and prime the immune system, thereby supporting the piglets’ robustness in the face of weaning-associated challenges.

## 2. Materials and Methods

### 2.1. Animals and Housing

This study was conducted in three rounds, with a total of 24 sows (Yorkshire x Landrace) mated with a Duroc boar. The sows were of 1–4 parity and tested as homozygotic carriers of the dominant gene coding for intestinal Enterotoxigenic *Escherichia coli* (ETEC) F18 fimbriae receptors [26]. The sows were transported from a commercial farm to the research facility on day 85 of the gestation, after which they were moved to the farrowing room on day 102 of the gestation. The sows and piglets were housed in one farrowing room, with eight loose farrowing pens (3.0 × 2.2 m) arranged in two rows of four pens. The pens had a partly slatted floor and were equipped with a covered creep area, an eating and drinking trough for the sow, and a nipple drinker for the piglets. Furthermore, the pens were designed with farrowing rails and a sloped wall. Physical contact between pigs in different pens was prevented by installing solid pen walls. The ventilation system was combi-diffuse ventilation, and the temperature was maintained at 20 °C during the first week, after which it was adjusted with 1 °C every week until a final temperature of 16 °C was reached. A heating lamp, placed in the covered creep area, was turned on before farrowing and kept on until seven days post farrowing. Additionally, extra heat for the piglets was provided for the first seven days through floor heating in the covered creep area.

Easystrø (Easy-AgriCare A/S, Denmark), which is heat-treated chopped straw, was used as bedding in the covered creep area for the first seven days after birth. Before farrowing, the sows were provided straw as bedding. After farrowing, the bedding was removed, but straw was allocated daily in a straw rack, and a rope was placed in each pen as an investigation and manipulation activity for the sows. Sows were fed a standard sow pelleted diet with an ingredient composition as described in Table 1. Sows were fed twice a day, and daily rations were allocated according to parity, stage of cycle and productivity. Piglets had no access to creep feed while suckling. All piglets were given iron supplementation on the first day of their lives.

After weaning on day 28 of age, litter mates were housed together in the same nursery pen. The nursery room contained eight pens (2.1 × 1.8 m) in two rows of four. Pens had partially slatted floors, and the concrete part of the floors had a covering and floor heating. The unit was neutral pressure ventilated and linked to temperature sensors. At the beginning of the study, the temperature was 24 °C. It was adjusted with ~1.5 °C every week, until a final temperature of 19 °C was reached. Nursery piglets were fed a nursery pelleted feed through two feeders with ad libitum access. The feed was a standard Danish nursery diet with an ingredient composition as described in Table 2. Fresh water was accessible through four drinking nipples. No straw was provided, but pigs had permanent access to ropes as an investigation and manipulation activity. Physical contact between pigs from different pens was prevented by installing solid pen walls. To prevent bacterial cross contamination between treatment groups, pigs in the control group were always handled before pigs in the probiotic group. When entering a pen or handling pigs, disposable gloves, shoe covers, aprons, and plastic sleeves were used. Plastic boxes assigned to each pen were used when weighing the pigs.

### 2.2. Preparation of Probiotic Inoculant

The experimental probiotic product consisted of four strains: *Bifidobacterium* (*B*.) longum subsp. *infantis*—228, *Bifidobacterium* (*B*.) breve—268, *Lacticaseibacillus* (*L*.) *rhamnosus* (formerly *Lactobacillus rhamnosus*)—994, and *Enterococcus* (*E*.) *lactis*—669 (formerly *Enterococcus faecium*). These four strains were selected based on a functional in vitro screening pipeline. The combination of the probiotic product was determined based on the beneficial and compatible attributes of the probiotic strains in regard to; barrier function, exclusion of ETEC F18 to intestinal epithelial cells, growth in porcine milk oligosaccharides and inhibitory effects towards ETEC F18 [25]. The probiotic product included the four different probiotic strains in a 1:1 ratio (1 × 10^9^ CFU/strain/pig/dose) blended with maltodextrin (0.35 g/pig/dose). The maltodextrin and freeze-dried probiotic mixture were blended beforehand and divided into portions stored in airtight bags; one bag per litter per dose. The placebo inoculant for the control group only included the maltodextrin (0.35 g/pig/dose). Placebo and probiotic mixtures were prepared just before each inoculation, by dissolving them in anaerobic phosphate buffered saline (PBS) (pH 7.4) (2 mL/pig/dose). The PBS solution was composed of sodium chloride, potassium chloride, dipotassium phosphate, monopotassium phosphate, L-Cysteine hydrochloride anhydrous, resazurine and reverse osmosis water. In round 1, the probiotic cocktail included 1 × 10^9^ CFU/dose *Faecalibacterium* (*F*.) *prausnitzii*—556 besides the other four strains, i.e., a total probiotic daily dosage of 5 × 10^9^ CFU/pig. However, *F. prausnitzii* was excluded from the probiotic product in round 2 and 3 due to difficulties with producing the strict anaerobic strain in the necessary scale. The effect of this was taken into account by including the interaction between round and probiotic treatment in the statistical analyses.

### 2.3. Experimental Design

At farrowing, 24 litters were randomly allocated to two treatments, and 168 piglets were included in each treatment group. Newborn piglets were orally inoculated with either placebo (control group) or probiotics (probiotic group). Inoculation was carried out at maximum 16 h after birth, once all piglets in the respective litter had been born. Placebo or probiotics were administered to piglets once a day at 9 a.m. for the first four days after birth, and subsequently every second day at 9 am until weaning on day 28. Inoculation was done using a Prima vaccinator device (Salfarm, Kolding, Denmark A/S) with a rubber tube. For the first four days, the rubber tube was dipped in apple juice before inoculation. Each piglet in the probiotic group was administered 4 × 10^9^ CFU/dose (or 5 × 10^9^ CFU/dose in round 1) dissolved in 2 mL anaerobic phosphate buffered saline and maltodextrin, whereas piglets in the control group were administered with the same volume of anaerobic phosphate buffered saline (to provide for the oxygen sensitive microbes) and maltodextrin. 48 h after farrowing, litters were standardized to 16 piglets, and five days after farrowing, litters were standardized to 14 piglets. In the standardization process, piglets which were excluded from the study were weak or previously treated with antibiotics. Cross-fostering was carried out, if necessary, within the first five days and only within treatment groups. All piglets were weaned on day 28 ± 2 of age, and no probiotics were administered post-weaning until the end of the experiment when the pigs were 50 days old. On the day of weaning, three and two piglets per litter from the control and probiotic group, respectively, were removed from this experimental study to be included in another study [27], and thus 8–9 piglets per litter were weaned. Seven days after weaning and after selecting two pigs per pen for slaughter, pens were adjusted to a maximum of five pigs by euthanizing pigs which were either weak or previously treated with antibiotics.

### 2.4. Registrations and Sample Collection

If piglets were treated with antibiotics, the reason was noted, and these piglets were not included in the subsequent collection of samples. Bodyweight of each individual pig was monitored every 7th day. After weaning, daily average feed intake per pen was likewise monitored. Occurrence of diarrhea in each pen was assessed daily during the entire experiment, according to the method of Toft and Pedersen [28]. Scores were: 1: Firm and shaped; 2: Soft and shaped; 3: Loose; 4: Watery. Diarrhea was defined as score 3 or 4, and diarrhea incidence (%) was calculated as number of pens with diarrhea (score 3 or 4) out of total number of days.

Three days after birth, three median piglets per litter were selected for collection of feces. These piglets were followed during the entire experiment by taking a rectal swap on day 3, 7, 14, 21, 28, 35, 42, and 50. Rectal swaps were collected using a cotton bud dipped in gel, and samples were kept on ice until storage. Samples were stored at −20 °C or −80 °C, depending on the further analysis. On day 28 (before weaning) and 35, a permeability challenge using Fluorescein isothiocyanate-dextran (FITC-d, 46944, Merck, Damstadt, Germany) was carried out. Piglets were orally administered with 1 mL per kg body weight FITC-d, and four hours later, a blood sample from the jugular vein was collected in heparinized vacutainers and kept on ice until storage. The samples were stored at −20 °C until further analysis.

On day 23–24 and day 35–36, two median pigs per litter were selected for blood sampling and for slaughter. Blood samples from the jugular vein were collected in EDTA-containing vacutainers for hematology analysis. Blood samples were analyzed immediately after collection. After blood sampling, pigs were euthanized using a captive bolt gun followed by bleeding. The gastrointestinal tract was removed, digesta content weighed and pH measured. The small intestine was divided into two (proximal and distal), and the colon was divided into three segments of equal length (proximal, mid, and distal). Digesta (stomach, proximal and distal small intestine, cecum, proximal, mid, and distal colon) from each of the two pigs per litter were pooled by taking the same amount from each pig and stored at −80 °C until further analysis. Mucosal samples were taken from the proximal and distal small intestine and proximal colon. Before sampling, the intestines were rinsed with sterile phosphate buffered saline several times to remove digesta and free-floating bacteria. Then, samples were collected by gently scraping off the mucosa from the epithelial layer by using a sterile glass microscope slide. Samples were kept in fluid nitrogen before being stored at −80 °C until further analysis.

### 2.5. Analytical Methods

The concentrations of DL-Lactic acid, branched-chained fatty acids, and short-chain fatty acids (SCFA) in feces and pooled digesta samples (proximal and distal small intestine, proximal, mid, and distal colon) were measured according to the method described by Canibe et al. [29]. The concentrations of agmatine, tyramine, cadaverine, and putrescine in feces and pooled digesta samples (distal small intestine, proximal, mid, and distal colon) were determined according to the method described by Poulsen et al. [5]. For dry matter analysis, freshly pooled digesta samples (stomach, proximal and distal small intestine, cecum, proximal, mid, and distal colon) were weighed before being stored at −20 °C and freeze dried.

For the host permeability test, FITC-d concentration in plasma was quantified via fluorescence spectroscopy by excitation at 480 nm and emission at 520 nm, using a standard curve of known concentrations. Immediately after collecting blood samples from the slaughtered pigs, hematological parameters (total white blood cells, neutrophils, lymphocytes, monocytes, eosinophils, red blood cells, packed cell volume (hematocrit), hemoglobin, reticulocytes and platelet counts) were analyzed in whole blood using a diagnostic health-monitoring tool (IDEXX ProCyte Dx^®,^, Sysmex Corporation, Lincolnshire, IL, USA).

For the gene expression analysis, total RNA was extracted from the distal small intestinal mucosal scrapings of the individual pigs (not pooled from two pigs) using the NucleoSpin RNA kit (Ref. 740955 Macherey-Nagel, Düren, Germany) including DNAse treatment. RNA was extracted following the instructions of the manufacturer with a pre-step homogenizing the samples for 2 × 2 min with a steel ball. Complementary DNA (cDNA) was synthesized from 1000 ng RNA using the High-Capacity cDNA Reverse Transcription Kit (Ref. 4368813, Applied Biosystems, Foster City, CA, USA) according to the manufacturer’s instructions. High-throughput quantitative real-time PCR was performed using the 192.24 dynamic array integrated fluidic circuits (Fluidigm, South San Fransisco, CA, USA) following methods previously described by Skovgaard et al. [30] with minor modifications, including 18 cycles of pre-amplification. qPCR was performed by combining 82 pre-amplified samples with 22 primer sets. Primer sequences and amplicon length for each assayed mRNA gene are listed in Table S1. Data were corrected for PCR efficiency for each primer assay individually and subsequently normalized using the average reference gene expression of three reference genes: Glyceraldehyde-3-Phosphate Dehydrogenase (GAPDH), Pepti-dylprolyl isomerase A (PPIA), and TATA-Box Binding Protein (TBP). The three reference genes were confirmed to be suitable endogenous reference genes, as they were not affected by the treatment. Normalized Cq values for each of the genes were converted to relative quantities by calculating 2(highest_assay_Cq–actual_sample_Cq), so that the sample with the highest Cq (lowest gene expression) was given a value of 1 and all other samples values > 1 as described by Brogaard et al. [31].

For 16S rRNA amplicon sequencing, feces, mucosa and digesta samples stored at −80 °C were thawed and weighed. DNA was extracted from 100 mg of feces and digesta samples using the E.Z.N.A.^®^ Stool DNA Kit (Omega Biotek, Norcross, GA, USA), following the instructions of the manufacturer, with the exception of including a steel ball for homogenization, until the step where the supernatant is aspirated. For mucosa samples, 12.5 mg from each of the two pigs per litter were pooled to extract DNA using the NucleoSpin Tissue DNA kit (Macherey-Nagel, Düren, Germany), according to the instructions of the manufacturer. DNA was diluted to 1 ng/μL using sterile water. The V3-V4 region of the 16S rRNA genes was amplified using the 341F and 806R primer (341F: 5′-CCTAYGGGRBGCASCAG-3′, 806R: 5′-GGACTACNNGGG-TATCTAAT-3′). All PCRs were conducted using the Phusion^®^ High-Fidelity PCR Master Mix (New England Biolabs, Inc., Ipswich, MA, USA). Agarose gel (2%) electrophoresis was performed to verify amplicon size using a 1X loading buffer (contained SYB green). Samples with bright main bands between 400 bp–450 bp were chosen for further analyses. PCR products were mixed at equal density ratios and purified with Qiagen Gel Extraction Kit (Qiagen, Hilden, Germany). Libraries were generated with the NEBNext^®^ UltraTM DNA Library Prep Kit for Illumina (New England Biolabs, Inc., Ipswich, MA, USA), quantified via Qubit and qPCR and submitted to sequencing. Sequencing was performed on an Illumina NovaSeq 6000 platform generating 2 × 250 bp paired-end sequence reads. Library preparation and sequencing was carried out by Novogene, Cambridge, UK.

### 2.6. Microbiome Data Analysis

Illumina MiSeq fastq files were processed using USEARCH (v.11.0) [32]. Raw reads were merged, trimmed, and quality filtered using the fstq_mergepairs and the fastq_filter scripts implemented in the USEARCH pipeline, as previously described by Krych et al. [33]. The UNOISE3 algorithm [34] with default settings was applied to denoise data, purge chimeric reads, and construct zero-radius operational taxonomic units (zOTU). Taxonomic assignment of zOTUs was performed with SINTAX [35] using the Greengenes (13.8) 16S rRNA gene collection reference database. Subsequent analysis steps were carried out using R (version 3.6.0) [36]. zOTUs unassigned at phylum or class level, zOTUs assigned as chloroplasts, mitochondria, *Cyanobacteria*, *Elusimicrobia*, *Planctomycetes*, or *Verrucomicrobia*, as well as zOTUs present in less than two samples and with a total abundance less than 0.001% across all samples were removed. Uneven sampling depth was normalized by rarefication to a read depth of 15,000 reads per sample using the Phyloseq package (version 1.30.0) [37], discarding 57 out of 665 samples (see rarefaction curve in Figure S1). If not stated differently, subsequent microbiome analyses were conducted for filtered and rarefied data subdivided based on sample type (feces, mucosa, digesta), sampling day (feces: d3, d7, d14, d21, d28, d35, d42, d50; mucosa and digesta: d23–24, d35–36) and sampling location within the gastrointestinal tract (mucosa and digesta: small intestine, proximal colon), separately. Microbial diversity analyses were performed using the packages Phyloseq [37] and Vegan [38]. For alpha diversity, observed number of zOTUs and the Shannon’s diversity index were calculated. Satisfaction of normality was tested using the Shapiro-Wilk test and effects of treatment and round on alpha diversity were investigated by linear mixed-effects models. Linear mixed-effects models were conducted using the lmer function implemented in the lme4 package (version 1.1-23) [39], with *treatment* and *round* as fixed effects and *sow* as random effect. For beta diversity, Bray-Curtis dissimilarity distances were estimated. Based on Bray-Curtis distances, a principal coordinate analysis (PCoA) was performed, and PCoA ordination plots were generated using the ggplot2 package (version 3.3.1) [40]. To investigate the effect of treatment group on beta diversity, a permutational multivariate analysis of variance (PERMANOVA) on Bray-Curtis distances was performed using the adonis function implemented in the Vegan package (version 2.5-6). Since adonis is not able to account for confounding factors, a potential confounding effect of *round* was tested by PERMANOVA in each of the sub-datasets. If *round* was found to be significant, the data were further divided based on *round*; if not, data for the three rounds were analyzed combined. Homogeneity of group dispersions (variance) was verified using the betadisper function implemented in Vegan, and a nested PERMANOVA on Bray-Curtis distances with *sow* nested within treatment group was carried out for each sub-dataset separately, using the function nested.npmanova on Bray-Curtis distances, based on the adonis algorithm implemented in the biodiversityR package (version 2.12-3) [41] and applying 999 permutations. Differential abundance analysis was carried out to identify community differences between treatment groups, using the DESeq2 package (version 1.2.6) [42] with filtered but not rarefied data. zOTU counts were normalized using the variance-stabilizing transformation approach implemented in DESeq2, and pseudo-counts of one were added to zero zOTU counts, as previously described by McMurdie and Holmes [37]. zOTUs were included in the results if the Log2 fold change >2 and if the adjusted *p*-value was ≤0.01. The ampvis2 package (version 2.5.0) [43] was used to generate heatmaps of the 15 most abundant families.

### 2.7. Statistical Analysis

All statistical analyses were carried out using R studio (version 3.6.0). QQ plots and the Shapiro-Wilk test were used to assess if the assumption of data being normally distributed was met. If this assumption was violated, data were log transformed or analyzed using a gamma distribution. The litter was considered as the experimental unit, and *sow* was thus included as a random factor. Day, round, and their interaction with treatment group were analyzed as fixed effects in all analyses. The linear mixed-effects model using the lmer function in the lme4 package was used to analyze FITC-d concentration in blood, pH in digesta as well as SCFA and biogenic amines in feces and digesta. The pen feces score was analyzed assuming a binomial distribution, by which score 1–2 was considered normal feces and score 3–4 was considered diarrhea. Hematology and digesta DM% data were analyzed using a gamma distribution within the glmer function. Gene expression data were log2 transformed to ensure a normal distribution prior to statistical analysis, using the lmer function in the lme4 package with *treatment*, *day*, and *round* as fixed effects and *sow* as random effect. GenEx ver 6 (MultiD, Gothenburg, Sweden) was used to calculate Spearman’s rank correlation coefficients and associated *p*-values (student-*t*-test). Spearman’s rank correlation coefficient was used to determine the correlation of (1) host gene expression in mucosa and DM%, SCFA and biogenic amines in digesta (2) expression of host and bacterial genes in mucosa, and (3) expression of host genes in mucosa and bacterial genes in digesta. Correlations were considered significant and illustrated in the results if the correlation was >0.5 and *p* < 0.01. In addition, a principal component analysis (PCA) was carried out on the log2 transformed gene expression data using the ggfortify package (version 0.4.9). For all analyses, the emmeans package was used to compare the effect of fixed factors. Data were given as least square means ± SEM. The significance was declared at *p* < 0.05, and *p* < 0.10 was considered a near-significant trend.

## 3. Results

Several statistical analyses showed a treatment-round interaction, but inconsistencies between round 1 and rounds 2 and 3 suggest that the inclusion of *F. prausnitzii* in the probiotic during round 1 had no clear impact on the results.

### 3.1. Piglet Health and Occurrence of Diarrhea

In the control and probiotic groups, 228 and 233 piglets, respectively, were born with an average birth weight of 1.4 ± 0.4 kg. On day 28, 120 control pigs were weaned with an average body weight of 8.4 ± 2.1 kg, and 101 probiotic pigs were weaned with an average body weight of 8.8 ± 1.9 kg. At the end of the experiment on day 50, piglets in the control and probiotic groups had an average body weight of 14.2 ± 3.5 kg and 13.2 ± 2.8 kg, respectively. Piglet mortality was mainly ascribed to crushing, i.e., 12 and 26 piglets in the control and probiotic group, respectively, whereas other causes were general weakness, joint infection or foot roots. The current experiment was not designed as a performance study, and since many of the piglets in each litter were selected and removed from the litter for slaughtering and for the parallel study, performance parameters were not analyzed and thus not presented.

34 and 31 piglets were treated with antibiotics in the control and probiotic group, respectively. Antibiotic treatment was predominantly due to joint infections and foot roots. Less than four piglets were treated with antibiotics due to diarrhea, and more than 95% of medical treatments were carried out before weaning. Five and three sows from the control and probiotic groups, respectively, were treated with antibiotics due to fever, lameness, udder infection, or farrowing difficulties. All analgesic and antibiotic treatments were provided intramuscularly.

**Figure 1:**
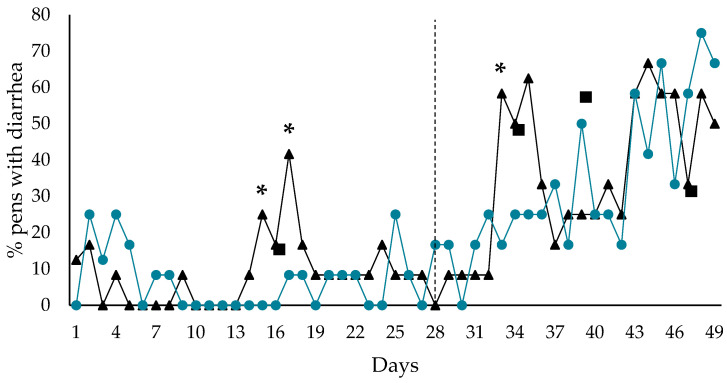
Effect of early probiotic inoculation on percentage of pens with diarrhea (score 3 and 4). Control ▲ (*n* = 12 pens), probiotic ● (*n* = 12 pens). The dotted line illustrates the day of weaning. * indicates statistical significance (*p* < 0.05), ■ indicates a trend towards statistical significance (*p* < 0.10).

Diarrhea occurrence, estimated for the entire experimental period, did not differ between control and probiotic pens (*p* = 0.82), with 10.1 and 9.6 days, respectively (Figure 1). However, the percentage of probiotic pens with diarrhea was lower compared with control pens on day 15, 17, and 33 (*p* < 0.05). In addition, the probiotic group tended to have a lower occurrence of pens with diarrhea compared with the control group on day 16 (*p* = 0.08) and 34 (*p* = 0.09). On the other hand, occurrence of diarrhea in the probiotic group tended to be higher than the control group on day 39 (*p* = 0.09) and 47 (*p* = 0.10).

### 3.2. Microbiota Composition in Gut Digesta and Mucosa, and in Feces

A microbiota analysis was carried out on the feces (*n* = 417), as well as the digesta and mucosa samples collected from the small intestine (digesta *n* = 73, mucosa *n* = 34) and proximal colon (digesta *n* = 44, mucosa *n* = 40). Sequencing of the 16S rRNA genes produced a total of 46,207,564 reads after quality filtration, ranging from 10 to 147,735 reads per sample. Rarefication to 15.000 reads per sample removed 57 samples but did not affect the number of detected zOTUs.

**Figure 2:**
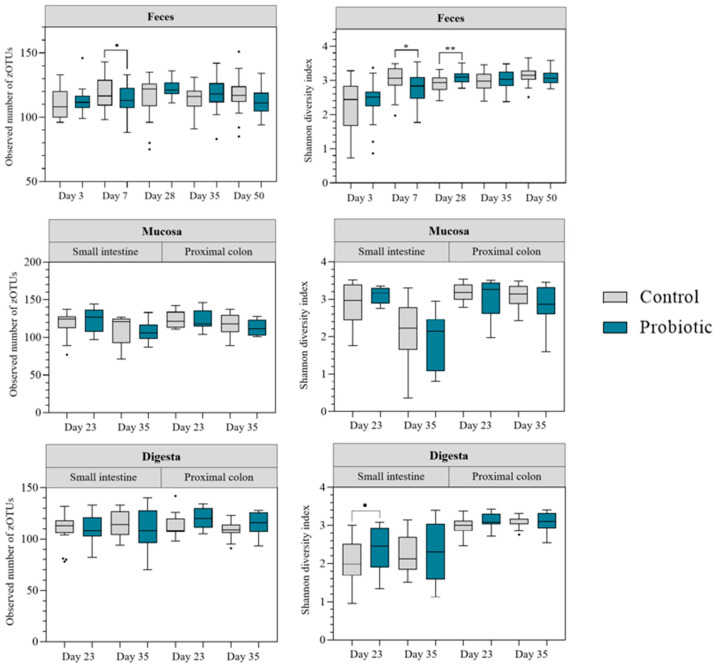
Observed number of zOTUs (**left**) and Shannon diversity index (**right**) of feces, mucosa, and digesta samples. Feces samples collected on day 3, 7, 28, 35 and 50 are shown. Mucosa and digesta samples were collected from the small intestine and proximal colon pre-weaning (day 23–24) and post-weaning (day 35–36). Pigs were orally administered with placebo or probiotics during suckling (day 1–28) and weaned on day 28. * and ** indicate statistical significance (*: *p* < 0.05 and **: *p* < 0.01), ■ indicates a trend towards statistical significance (*p* < 0.10).

Alpha diversity indices were compared for rarefied zOTU counts (Figure 2). For feces samples, the Shannon index increased over time. The observed number of zOTUs (microbial richness) was stabler throughout the experiment. These trends were the same for both treatment groups. Differences between groups were observed on day 7 and day 28. On day 7, the Shannon diversity index was lower in the probiotic group (*p* = 0.01) and comparable tendencies were also observed for the microbial richness (*p* < 0.10). On day 28, in contrast, the Shannon diversity index was higher in the probiotic group (*p* = 0.006). Alpha diversity in mucosal samples was not affected by probiotic inoculation. However, in the small intestine alpha diversity decreased post-weaning, irrespective of treatment group (*p* < 0.05). For digesta samples, the Shannon diversity was higher in samples collected in the proximal colon than in samples from the small intestine, irrespective of treatment group (*p* < 0.05). However, for samples collected in the small intestine the Shannon diversity index tended to be higher in the probiotic group on day 23–24 (*p* = 0.06).

**Figure 3:**
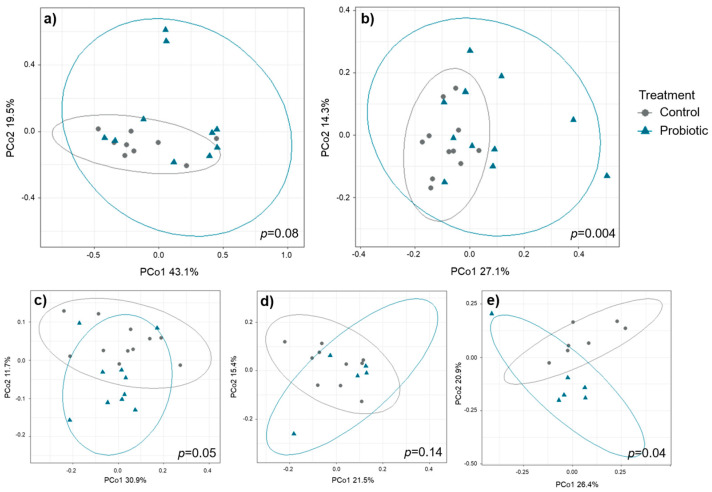
Principal coordinates analysis (PCoA) of Bray-Curtis dissimilarity between the control and probiotic group on day 35. Bray-Curtis distance metrics were used to compare the composition of the microbiota between the two treatment groups in (**a**) small intestinal mucosa, (**b**) digesta of the proximal colon, and feces in *round* (**c**) 1, (**d**) 2, and (**e**) 3. Nested permutational multivariate analysis of variance (PERMANOVA) on Bray-Curtis distance metrics with sow ID nested with treatment group was carried out using 999 permutations to test for the significance of the clustering pattern. *p*-values for the effect of probiotic treatment are illustrated. *p* < 0.05 was considered significant whereas *p* < 0.10 was considered a statistical tendency.

Beta diversity did not differ pre-weaning but showed differences between the two treatment groups after cessation of probiotic administration on day 35 (Figure 3). The strongest differences between treatments on day 35 were observed in digesta samples collected from the proximal colon (*p* = 0.004) followed by feces samples in round 3 (*p* = 0.04) and round 1 (*p* = 0.05). Compared to the other sample types, *round* had a profound impact on beta diversity in feces samples, hence, PERMANOVA analyses were conducted for each of the three rounds separately (round 1: *p* = 0.05, round 2: *p* = 0.14, round 3: *p* = 0.04). For mucosal samples, microbial beta diversity tended to differ between treatments in the samples collected from the small intestine (*p* = 0.08) but not in samples collected from the proximal colon. Differences in beta diversity on day 35 were not associated with litter origin (Figure S2).

The heatmap of the top 15 families in feces illustrates that the abundance of Enterobacteriaceae was lower in the probiotic group on day 3, whereas abundances of *Bacteroidaceae* and *Fusobacteriaceae* were higher (Figure S3). Until two weeks of age, the probiotic group had a higher abundance of *Lactobacillaceae* and a lower abundance of *Bacteroidaceae* in feces. In small intestinal mucosal samples collected pre-weaning, the probiotic group had a higher abundance of *Veillonellaceae* and a lower abundance of *Helicobacteraceae* (Figure S4). Observed differences in *Helicobacteraceae* abundance in small intestinal mucosal samples persisted post-weaning. Interestingly, in mucosa samples collected in the proximal colon, *Helicobacteraceae* were more abundant in probiotic pigs, at least post-weaning. In digesta samples, the *Helicobacteraceae* family was not part of the top 15 most abundant families (Figure S5). In small intestinal mucosa samples collected post-weaning, probiotic pigs had a higher proportion of *Enterobacteriaceae* and *Lactobacillaceae*. On the contrary, *Enterobacteriaceae* abundance in small intestinal digesta was lower in the probiotic group post-weaning. *Spirochaetaceae* was found in higher proportions in digesta from the proximal colon of probiotic pigs post-weaning.

**Figure 4:**
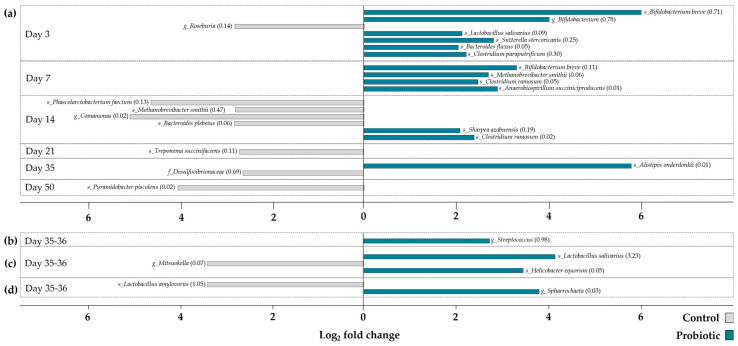
Differential abundance analysis showing zOTUs significantly discriminating the control and probiotic treatment groups in (**a**) feces, (**b**) small intestinal mucosa on day 35–36, (**c**) small intestinal digesta on day 35–36, and (**d**) digesta from proximal colon on day 35–36. Log2 fold change in the control group (**left**) and the probiotic group (**right**) is illustrated. Mean relative abundance is presented in parenthesis following the zOTU’s taxonomic name. zOTUs were defined as differentially abundant if they had a log2 fold change >2 and an adjusted *p* ≤ 0.01. Only differentially abundant zOTUs are presented.

zOTUs that significantly discriminated the two different treatment groups were identified by differential abundance analysis (Figure 4). In total, 27 differentially abundant zOTUs were identified. The majority of differentially abundant zOTUs was detected in feces samples collected pre-weaning (*n* = 18), followed by feces samples collected post-weaning (*n* = 3). Number of differentially abundant zOTUs in mucosal and digesta samples were only found in samples collected after weaning. Overall, zOTUs discriminating the control and probiotic groups were found in higher abundance in feces of probiotic pigs during the first week of life. Afterwards, zOTUs differentiating the two treatment groups were more abundant in the feces of the control pigs. Looking at zOTUs that significantly discriminate the microbial community in probiotic pigs from control pigs, *B. breve*, *L. salivarius*, as well as *C. ramosum* stand out by reoccurring on different days or in different samples (feces and mucosa). None of the zOTUs significantly more abundant in the control pigs were found to reoccur.

### 3.3. Gene Expression in Small Intestinal Mucosa and Correlation Analyses

**Figure 5:**
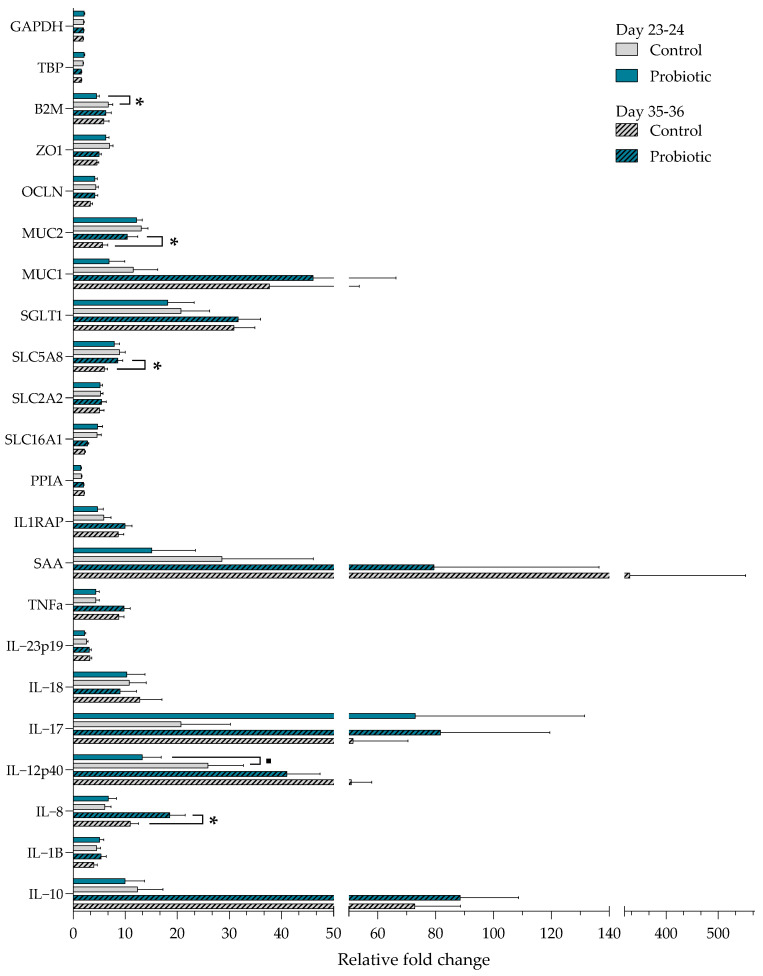
Fold change of the small intestinal mucosal gene expression in control and probiotic pigs on day 23–24 and day 35–36. * indicates the statistical significance between treatment groups (*p* < 0.05), ■ indicates a trend towards statistical significance (*p* < 0.10), determined by a mixed model. Number of samples: Control d23–24 = 22, d35–36 = 22. Probiotic d23–24 = 20, d35–36 = 18.

PCA plots of all gene expression data discriminating between samples belonging to different treatment groups or time points are shown in Figure S6. The figure suggests that the overall gene expression differs between pre- and post-weaning but not between treatments. Gene expression patterns between the probiotic and control pigs indicate that most of the analyzed genes in pigs before weaning were expressed at a lower numerical level in the small intestinal mucosa of the probiotic pigs (Figure 5). The expression of B2M was lower in the probiotic group pre-weaning (*p* = 0.02), and a tendency for reduced expression of the pro-inflammatory cytokine IL-12p40 was also observed (*p* = 0.06). Post-weaning, the probiotic pigs had an overall higher expression of most genes. The expression of MUC2 (*p* = 0.02), SLC5A8 (*p* = 0.003), and IL-8 (*p* = 0.01) was upregulated in probiotic pigs post-weaning. Compared with the other genes tested, SAA was expressed at a high level in control pigs both pre- and post-weaning, whereas IL-17 was upregulated in the probiotic pigs pre- and post-weaning compared with the control pigs. A significant interaction between day and treatment was found for B2M, MUC2, OCLN, and SLC5A8 (*p* < 0.05). All four genes were upregulated in the control pigs before weaning as compared to after weaning (*p* < 0.05).

**Figure 6:**
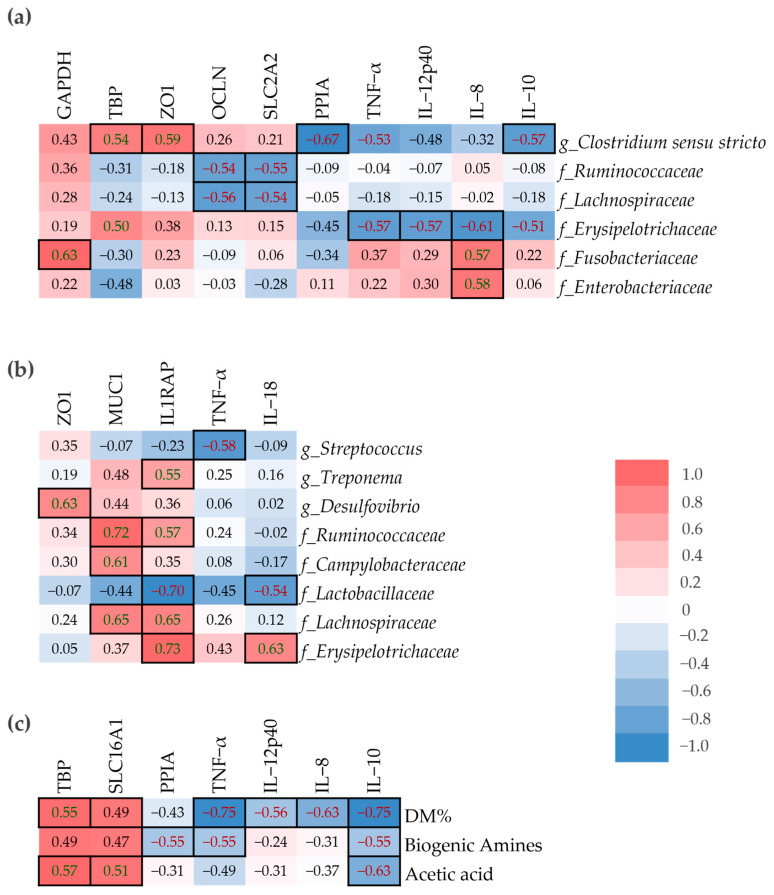
Correlation between small intestinal mucosa genes and the abundance of small intestinal microbiota (at zOTU level) (**a**) in mucosa (*n* = 22), (**b**) in digesta (*n* = 20), and (**c**) dry matter percentage, concentration of biogenic amines and acetic acid in small intestinal digesta (*n* = 43). The color is assigned according to Spearman’s rank correlation coefficient distribution; red presents positive correlation, blue presents negative correlation. Bold frame indicates statistically significant correlation, which was appointed when *p* < 0.01 and correlation coefficient <−0.5 or >0.5.

A spearman’s correlation analysis was performed to determine the relationship between host-gene expression in small intestinal mucosa samples and microbial composition in mucosa and digesta samples, as well as DM%, SCFA and biogenic amines in digesta (Figure 6). The *Erysipelotrichaeceae* family was positively correlated with IL-8 in digesta, but the correlation was negative in mucosa (Figure 6a,b). *Ruminococcaceae* and *Lachnospiraceae* abundance in the small intestinal mucosa were negatively correlated with OCLN and SLC2A2, yet their abundance in small intestinal digesta was positively correlated with MUC1 and IL1RAP. IL-8 was positively correlated with *Fusobacteraceae* and *Enterobacteriaceae* in mucosa and negatively correlated with *Lactobacillaceae* in digesta. The dry matter percentage of small intestinal digesta was negatively correlated with the pro-inflammatory cytokines TNF-α, IL-12p40, and IL-8, but also the anti-inflammatory cytokine IL-10 (Figure 6c). On the other hand, both TBP and SLC16A1 were positively correlated with DM%.

### 3.4. Microbial Metabolites, DM% and pH

#### 3.4.1. Feces

**Figure 7:**
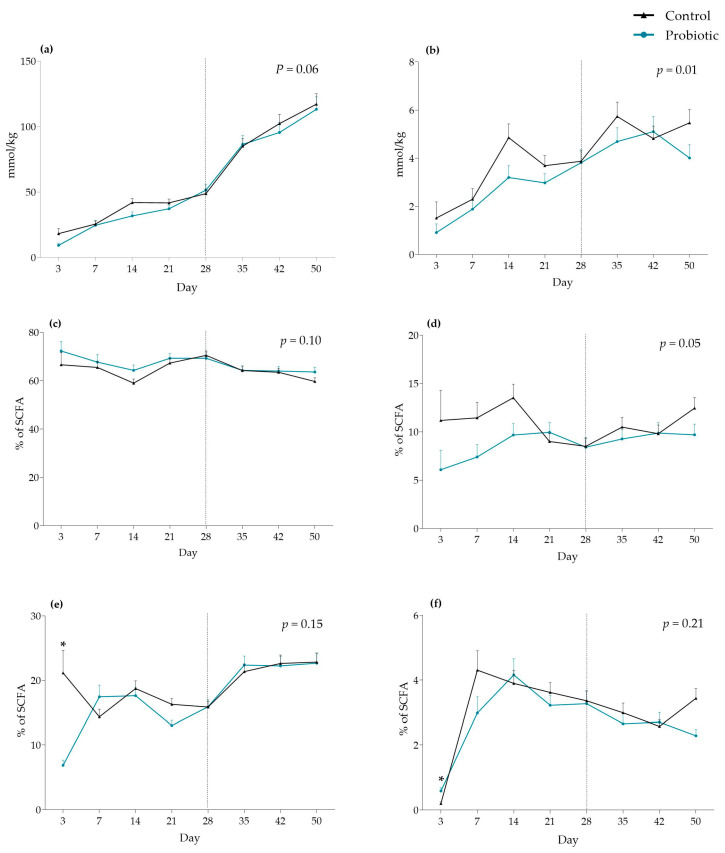
Effect of early probiotic inoculation on (**a**) short-chain fatty acids (SCFA), (**b**) isobutyric and isovaleric acids, and percent (**c**) acetic, (**d**) butyric, (**e**) propionic, and (**f**) valeric acid of total SCFA concentration in feces (mmol/kg). The dotted line illustrates the day of weaning. Values are presented as least square means and SE, and the *p*-value for the effect of probiotic treatment is stated. * indicates a statistical significance (*p* < 0.05) between treatments on a specific day. Number of samples: Control d3 = 3, d7 = 12, d14 = 21, d21 = 26, d28 = 27, d35 = 27, d42 = 27, d50 = 28. Probiotic d3 = 6, d7 = 8, d14 = 15, d21 = 22, d28 = 18, d35 = 21, d42 = 20, d50 = 18.

The probiotic group tended to have lower concentrations of SCFA in feces compared with the control group during the entire experimental period (*p* = 0.06) (Figure 7a). An interaction between treatment and round was found, indicative of SCFA concentration in feces being significantly higher in the probiotic group during round 3 compared with round 1 (*p* = 0.03) and 2 (*p* < 0.001). Additionally, the total SCFA concentration in feces was significantly lower in probiotic pigs during round 2 (*p* = 0.01). The concentration of branched-chain fatty acids (isobutyric and isovaleric acid) in feces was significantly lower in the probiotic group compared with the control group (*p* = 0.01) (Figure 7b). The percentage of formic acid of total SCFA was low (<2%) in both treatment groups; thus it was not included in the illustrated results. The probiotic group tended to have a higher proportion of acetic acid (*p* = 0.10) (Figure 7c) and a lower proportion of butyric acid (*p* = 0.05) (Figure 7d) in feces. An interaction between day and treatment was observed in the percent of propionic acid, driven by the percent being significantly lower in the probiotic group compared with the control group on day 3 (*p* < 0.001). Likewise, the probiotic group had a significantly higher proportion of valeric acid on day 3 compared with the control group (*p* = 0.001).

No significant effect of the treatment on the concentration of biogenic amines in feces pre- and post-weaning was found (Figure S7). In general, concentrations were high on day 3 and 7 but decreased until weaning on day 28. Post-weaning, the concentration of biogenic amines started to increase again.

#### 3.4.2. Digesta

Probiotic treatment did not significantly impact the concentration of organic acids along the gastrointestinal tract (Tables S2 and S3). Yet, the probiotic pigs tended to have a lower proportion of valeric acid in colonic content post-weaning (*p* = 0.06). The concentration of SCFA was significantly higher post-weaning compared with pre-weaning in both treatment groups (*p* < 0.001). Probiotic inoculation tended to increase the concentration of agmatine in intestinal content pre-weaning (*p* = 0.10), and the concentration of tyramine was found to be lower in the distal small intestine of probiotic pigs pre-weaning (*p* = 0.04) (Table S4). Post-weaning, the concentration of cadaverine tended to be lower in the probiotic group (*p* = 0.09), and the concentration of agmatine was higher in the mid and distal colon of probiotic pigs post-weaning (*p* = 0.04) (Table S5).

Probiotic inoculation did not influence pH in any of the segments pre-weaning (Table S6). However, the post-weaning pH was significantly higher in the stomach of probiotic pigs (*p* = 0.005) (Table S7). No impact of probiotic inoculation was found on pH in any of the other intestinal segments post-weaning. The digesta dry matter percentage was not affected by probiotic inoculation (Tables S8 and S9). It was significantly affected by day and segment in both treatment groups, and DM% was higher in all intestinal segments before weaning.

### 3.5. Permeability Challenge and Hematological Parameters

**Figure 8:**
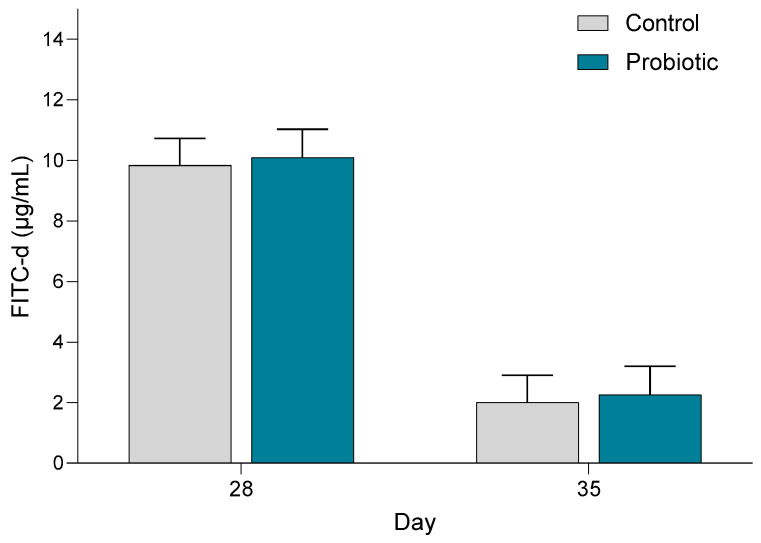
Fluorescein isothiocyanate-dextran (FITC-d) concentration (µg/mL) in the blood 4 h after oral administration of FITC-d pre- and post-weaning in piglets in the control and probiotic groups. Number of samples: Control d28 = 11, d35 = 11; Probiotic d28 = 9, d35 = 9.

The concentration of FITC-d in the blood was significantly higher in both treatment groups on day 28 (before weaning) (*p* < 0.001) compared with day 35 (Figure 8). Probiotic inoculation had no significant impact on the FITC-d concentration in blood.

The probiotic group had a significantly higher concentration of reticulocytes in the blood post-weaning (*p* = 0.02) (Table S10). The rest of the hematological parameters were not influenced by the probiotic treatment (*p* > 0.05). Hematocrit, red blood cells, hemoglobin and total white blood cells increased from pre- to post-weaning (*p* < 0.001), whereas reticulocytes and platelets decreased (*p* < 0.01). The concentration of white blood cells increased with 70% and 35% from pre- to post-weaning in the control and probiotic groups, respectively (*p* = 0.11).

## 4. Discussion

This study hypothesized that inoculating a multi-species probiotic product consisting of *L. rhamnosus*, *E. lactis*, *B. longum* subsp. *infantis*, and *B. breve* in newborn piglets and during suckling would prime the gut microbiota in a beneficial way and accelerate the development of the immune system. Studies investigating the effect of early probiotic administration to piglets found that the beneficial effects of probiotics on intestinal morphology and immunity slowly diminished post-weaning and after cessation of probiotic administration [44,45]. In our study, early probiotic administration exerted the highest effect after weaning, when the probiotics were no longer provided, which was expressed as significant differences in the beta diversity of the microbial community in both feces and intestinal content and tissue, as well as a shift in gene expression in the small intestinal mucosa. However, the large variation between pigs, as illustrated in the PCoA plots, may indicate that the administered probiotics influenced each individual piglet differently.

In the current study, alpha diversity was found to be lower in feces from probiotic pigs on day 7. Yet, prior to weaning on day 23 and 28 the pigs supplemented with probiotics exerted higher Shannon diversity in feces and the small intestinal digesta. Several studies assessed the effect of early probiotic inoculation to pigs on the gut microbiome community and found no effect on gut bacterial microbiota richness and diversity [46,47,48,49]. In contrast to the alpha diversity results, beta diversity of the overall microbial composition was not significantly affected during the administration of the multi-species probiotic but showed significant changes by day 35, i.e., after the probiotic administration had ended. According to Lewis et al. [21], supplementation of *B. lactis* pre- and post-weaning influenced metabolic reactions such as urinary formate excretion of the host rather than the composition of the gut microbiota; therefore, it would have been interesting to look further into host metabolism in the current study. Indeed, the differential abundance analysis underlined specific zOTUs that were significantly different between the two treatment groups. Especially on day 3 and 7, several zOTUs were significantly more abundant in feces from probiotic pigs, including species of *Ligilactobacillus* and *Bifidobacterium*. Both species are considered beneficial for the host due to their antibacterial and immunomodulatory properties [50]. *B. breve*, which was one of the species inoculated in the probiotic product, was significantly more abundant in the probiotic group on both day 3 and 7. Supplementation of *B. breve* early in life has previously shown to improve the development of mucosal immunity in rats [51]. *L. salivarius* was significantly more abundant in feces taken on day 3 and in the small intestinal digesta on day 35–36 in pigs supplemented with probiotics. The *L. salivarius* species has previously been evaluated as an early inoculation probiotic for piglets [52]. These authors established that *L. salivarius* improved intestinal health and reduced diarrhea incidence in suckling piglets challenged with ETEC F4. Early inoculation of the probiotic mixture in this study likely provided an early competitive advantage to the supplemented strains or to taxa supported by them, such as *B. breve* and *L. salivarius*, which were found in higher abundance in probiotic pigs. These taxa are known for their ability to colonize the neonatal gut, utilize milk-derived oligosaccharides, and create a favorable environment for the establishment of a beneficial microbial community [21]. By shaping the early microbiota trajectory, the probiotic inoculation may have promoted a more stable and health-associated microbial profile that persisted beyond the supplementation period. This early modulation could have helped suppress the proliferation of opportunistic pathogens and enhanced mucosal immune maturation, as reflected by the differences in gene expression profiles observed post-weaning.

The probiotic pigs had a higher expression of the gene MUC2 post-weaning. Even though MUC2, which is an important part of the intestinal barrier function, was upregulated post-weaning in the probiotic pigs, intestinal permeability was, according to the FITC-d permeability test, not influenced by early probiotic inoculation. We hypothesized that gastrointestinal leakage would be higher post-weaning; however, our results showed the opposite. To our knowledge, no other studies have measured intestinal permeability using the FITC-d method in pre- and post-weaning pigs. Perhaps the higher permeability pre-weaning is attributable to a more immature intestinal epithelial barrier, yet several studies have demonstrated decreased intestinal morphology due to weaning of the pigs [53,54].

The acute phase protein SAA was numerically upregulated in non-probiotic supplemented pigs, especially post-weaning. It is well established that SAA levels are significantly elevated after weaning [55], and that an elevation of acute phase proteins including SAA is an early sign of disease [56]. The intestinal mucosa has been proposed to be the production site of acute phase proteins, such as SAA, both in response to local and systemic inflammation [57]. In combination with a lower expression of SAA in the small intestinal mucosa of pigs in the probiotic group, there were fewer pens with diarrhea following weaning in this group compared to pigs not administered with probiotics. In agreement with our study, Liu et al. [58] demonstrated a reduced incidence of diarrhea after weaning and cessation of *E. faecalis* and *L. casei* administration. Electrophoresis of DGGE with a DNA marker for the two administered probiotic strains indicated that the administered strains did not establish themselves in the gastro-intestinal tract [58]. Hence, previous and current results indicate that piglets supplemented with probiotics early in life and during suckling may be better at overcoming the weaning process, not by the early inoculated strains establishing themselves in the gastro-intestinal tract, but perhaps by a more accelerated maturation of the mucosal immune system. The percentwise lower increase in total white blood cells from pre- to post-weaning in probiotic pigs may likewise indicate that the probiotic pigs were less affected by weaning stress [59], possibly due to a more efficient mucosal immune response. In addition, Lewis et al. [21] proposed that early life events causing e.g., elevated expression of IL-17 may drive long-term immunomodulation, possibly expressed by an increased local mucosal immune response due to early probiotic priming. Such reactivity is important when the pig is challenged, for example at weaning. However, over-responsiveness of the immune system can also be detrimental for health and performance during non-challenged situations [60]. According to Cox et al. [61] and Benis et al. [62], early life crosstalk between gut microbes and the host drives the long-term immune and metabolic programming, yet not by persistent changes in the gut microbiota composition. In fact, Benis et al. [62] proposed that to observe the effect of an early life intervention, the pig should later in life be exposed to stressors like pathogen challenges, or as in our study, weaning. The findings from Cox et al. [61] and Benis et al. [62] may thus be part of explaining the results from our study.

Fermentation of dietary fibers by the gut microbiota results in SCFAs as metabolic end-products, which are mostly absorbed, and the non-absorbed fraction, excreted in feces [63]. In this study, probiotic inoculation decreased the concentration of SCFA and branched chain fatty acids in feces. This was not the case in the small intestinal and colonic content, where the levels did not differ between treatment groups. In agreement with the latter, Hou et al. [20] demonstrated that early inoculation of *L. reuteri* to piglets had no effect on the SCFA concentration in colonic content on day 7, 14, or 21. These results were found regardless of the probiotic being inoculated daily for the first four days after birth or every 4th day from day 1 to 17. SCFAs are important metabolites for maintenance of intestinal homeostasis. High levels of SCFAs are generally considered as beneficial for gut health [64]. However, a recent study suggests that high fecal concentrations of SCFA in fact are associated with dysbiosis and high permeability of the gut [63]. The authors hypothesized that high fecal concentrations of SCFA may be due to less efficient absorption and utilization of these metabolites. Whether the increased SCFA concentrations observed in feces of non-probiotic supplemented pigs in the present study were the result of a reduced absorption efficiency is not clear, and future research should investigate the relationship between intestinal and fecal levels of SCFA and other metabolites.

The level of biogenic amines in intestinal content, but not in feces, was influenced by probiotic inoculation. Both pre- and post-weaning, agmatine, which is formed from microbial decarboxylation of the amino acid L-arginine, was found in higher concentrations in intestinal content from probiotic pigs. Agmatine has been shown to be an important biological compound involved in apoptosis and inflammation processes in different animal species [65]. To our knowledge, the importance of this metabolite in pigs has not yet been established. According to Porter and Kenworthy et al. [66], high levels of cadaverine, especially in the small intestine, is associated with diarrhea in weaned pigs. In this study, non-probiotic supplemented pigs tended to have higher concentrations of cadaverine in intestinal content post-weaning, which may have contributed to the higher prevalence of diarrhea which was observed one-week post-weaning. Another interesting observation irrespective of probiotic treatment was the overall high concentrations of biogenic amines on day 3 compared with later in life, which is consistent with results reported by Poulsen et al. [5]. These high concentrations are probably a result of the specific microbiota composition found in newborn piglets, with high levels of groups like *Enterobacteriaceae* and *Clostridiaceae* [67], as well as the substrate availability in the form of colostrum, i.e., high protein content [68]. It can be speculated whether high levels of biogenic amines are crucial for intestinal maturation.

For most response parameters analyzed, *round* was identified as having a significant effect. Batch or round effects are often detected in animal studies, which adds complexity when concluding on the effect of the tested intervention. Understanding the sources of variation would be helpful when drawing conclusions, and perhaps also make the findings more applicable for the industry. In this study, only effects that were consistent across all three rounds were emphasized.

## 5. Conclusions

This study demonstrates that early-life administration of a multi-species probiotic to piglets can lead to lasting modifications in the gut microbiota composition and immune responses, extending beyond the weaning period. Notably, probiotic-treated piglets exhibited increased abundance of beneficial bacteria such as *B. breve* and *L. salivarius*, alongside an elevated expression of immune markers, including MUC2, IL-8, and IL-17 post-weaning. These changes were associated with a reduced incidence of post-weaning diarrhea. The findings suggest that early probiotic intervention may enhance resilience during critical developmental stages, offering a promising strategy to improve piglet health and potentially contribute to more prudent use of antimicrobial treatments in swine production.

It would be of great interest to assess the impact of early probiotic inoculation on pigs under more challenging conditions than those present in the current study, such as environments with higher pathogen loads or increased stocking densities. Furthermore, long-term follow-ups of probiotic-treated pigs could help clarify the significance of the observed short-term effects for animal health and performance later in life.

## Figures and Tables

**Table 1:** Ingredient composition of the sow diet.

Ingredients	%
Barley	41.13
Wheat	25.00
Soybean meal	13.30
Oats	5.00
Wheat bran	5.00
Sunflower meal	2.00
Soybean hulls	2.00
Dried beet pulp	2.00
Calcium carbonate, chalk	0.90
Palm fatty acid distillate	0.90
Monocalcium phosphate	0.65
Vitamin and micromineral premixture	0.55
Sodium chloride	0.53
L-Lysine sulphate	0.43
Axtra XB (enzyme combination)	0.20
E vitamin mixed in wheat middling	0.18
Threonine 98%	0.11
DL-Methionine	0.06
Phytase enzyme (E4a24)	0.06

**Table 2:** Ingredient composition of the nursery diet.

Ingredients	%
Wheat	53.21
Barley	21.00
Soybean meal	8.00
ViloSoy, soy protein	6.75
Potato protein, Protastar	3.50
Palm fatty acid distillate	1.98
Calcium carbonate, chalk	1.25
Monocalcium phosphate	1.06
Fish meal	1.00
Lysine sulphate 70	0.67
Sugar beet molasses	0.50
Vitamin premixture	0.40
Sodium chloride	0.36
Threonine 98%	0.12
DL-Methionine 98	0.11
Tryptophane 99	0.04
Ronozyme HiPhos (phytase)	0.03
Valine L 96.5	0.02

## Data Availability

BioSample metadata are available in the NCBI BioSample database (http://www.ncbi.nlm.nih.gov/biosample/, accessed on 12 July 2024) under accession number SAMN14600997.

## Supplementary Materials

The following supporting information can be downloaded at: https://www.mdpi.com/article/10.3390/microorganisms13061292/s1, Figure S1. Rarefaction curve of all samples; Figure S2. Principal coordinates analysis (PCoA) of Bray-Curtis dissimilarity between the control and probiotic group on day 35; Figure S3. Heatmap of fecal samples collected at day 3, 7, 14, 21, 28, 35, 42 and 50 from pigs administered placebo (control) or probiotics during suckling; Figure S4. Heatmap of mucosal samples collected from the small intestine and ascending colon (CO1) at day 23–24 (pre-weaning) and day 35–36 (post-weaning); Figure S5. Heatmap of digesta samples collected from the small intestine and ascending colon (CO1) at day 23–24 (pre-weaning) and day 35–36 (post-weaning); Figure S6. Principle component analysis (PCA) plot on Log2 transformed gene expression data discriminated between samples belonging to (a) the two different treatment groups (control and probiotic), or (b) the two different time points (day 23–24 or day 35–36); Figure S7. Effect of early probiotic inoculation on the biogenic amines (a) tyramine, (b) agmatine, (c) putrescine, (d) cadaverine, and (e) the total concentration of biogenic amines in feces; Table S1. mRNA primer sequences and amplicon length (F: Forward, R: Reverse); Table S2. Effect of early inoculation of probiotics on DL-lactic acid, short-chain fatty acids and branched fatty acids in small intestinal (SI) and colonic (CO) intestinal content on day 23–24 pre-weaning; Table S3. Effect of early inoculation of probiotics on DL-lactic acid, short-chain fatty acids and branched fatty acids in small intestinal (SI) and colonic (CO) intestinal content on day 35–36 post-weaning; Table S4. Effect of early inoculation of probiotics on biogenic amines in small intestinal (SI) and colonic (CO) intestinal content on day 23–24 pre-weaning; Table S5. Effect of early inoculation of probiotics on biogenic amines in small intestinal (SI) and colonic (CO) intestinal content on day 35–36 post-weaning; Table S6. Effect of early inoculation of probiotics on pH in content from the stomach, small intestine (SI), caecum, and colon (CO) on day 23–24 pre-weaning; Table S7. Effect of early inoculation of probiotics on pH in content from the stomach, small intestine (SI), caecum, and colon (CO) on day 35–36 post-weaning; Table S8. Effect of early inoculation of probiotics on dry matter percentage in content from the stomach, small intestine (SI), caecum, and colon (CO) on day 23–24 pre-weaning (DM%); Table S9. Effect of early inoculation of probiotics on dry matter percentage in content from the stomach, small intestine (SI), caecum, and colon (CO) on day 35–36 post-weaning (DM%); Table S10. Effect of early probiotic inoculation on hematology parameters in blood on day 23–24 and 35–36.

## Abbreviations

The following abbreviations are used in this manuscript:

B2M	Beta-2-Microglobulin
CFU	Colony forming unit
DM%	Dry matter percentage
ETEC	Enterotoxigenic *Escherichia coli*
FITC-d	Fluorescein isothiocyanate-dextran
GAPDH	Glyceraldehyde-3-Phosphate Dehydrogenase
IL-1B	Interleukin 1 Beta
IL1RAP	Interleukin 1 Receptor Accessory Protein
IL-8	Interleukin 8
IL-10	Interleukin 10
IL-12p40	Interleukin 12 subunit beta
IL-17	Interleukin 17
IL-18	Interleukin 18
IL-23p19	Interleukin 23 subunit alpha
MUC1	Mucin 1, cell surface associated
MUC2	Mucin 2, oligomeric mucus gel-forming
OCLN	Occludin
PCA	Polymerase Chain Reaction
qPCR	quantitative real-time PCR
PPIA	Peptidylprolyl Isomerase A
SAA	Serum Amyloid A
SCFA	Short-chain fatty acid
SLC5A1	Solute Carrier Family 2
SLC2A2	Solute Carrier Family 2
SLC5A8	Sodium-coupled monocarboxylate transporter 1
SLC16A1	Solute Carrier Family 16 Member 1
TBP	TATA-binding protein
TNFα	Tumor Necrosis Factor alpha
ZO1	Zonula occludens-1
zOTU	zero-radius operational taxonomic units

## References

[1] Fairbrother J.M., Nadeau É., Gyles C.L. (2005). Escherichia coli in postweaning diarrhea in pigs: An update on bacterial types, pathogenesis, and prevention strategies. Anim. Heal. Res. Rev..

[2] Docic M., Bilkei G. (2003). Differences in antibiotic resistance in Escherichia coli, isolated from East-European swine herds with or without prophylactic use of antibiotics. J. Vet. Med. Ser. B.

[3] Gaskins H.R., Kelley K.W., Varley M.A. (1995). Immunology and Neonatal Mortality. The Neonatal Pig—Development and Survival.

[4] Frese S.A., Parker K., Calvert C.C., Mills D.A. (2015). Diet shapes the gut microbiome of pigs during nursing and weaning. Microbiome.

[5] Poulsen A.-S.R., de Jonge N., Nielsen J.L., Højberg O., Lauridsen C., Cutting S.M., Canibe N. (2018). Impact of Bacillus spp. spores and gentamicin on the gastrointestinal microbiota of suckling and newly weaned piglets. PLoS ONE.

[6] Motta V., Luise D., Bosi P., Trevisi P. (2019). Faecal microbiota shift during weaning transition in piglets and evaluation of AO blood types as shaping factor for the bacterial community profile. PLoS ONE.

[7] Dou S., Gadonna-Widehem P., Rome V., Hamoudi D., Rhazi L., Lakhal L., Larcher T., Bahi-Jaber N., Pinon-Quintana A., Guyonvarch A. (2017). Characterisation of early-life fecal microbiota in susceptible and healthy pigs to post-weaning diarrhoea. PLoS ONE.

[8] Merrifield C.A., Lewis M.C., Berger B., Cloarec O., Heinzmann S.S., Charton F., Krause L., Levin N.S., Duncker S., Mercenier A. (2016). Neonatal environment exerts a sustained influence on the development of the intestinal microbiota and metabolic phenotype. ISME J..

[9] Stokholm J., Thorsen J., Blaser M.J., Rasmussen M.A., Hjelmsø M., Shah S., Christensen E.D., Chawes B.L., Bønnelykke K., Brix S. (2020). Delivery mode and gut microbial changes correlate with an increased risk of childhood asthma. Sci. Transl. Med..

[10] Mulder I.E., Schmidt B., Lewis M., Delday M., Stokes C.R., Bailey M., Aminov R.I., Gill B.P., Pluske J.R., Mayer C.-D. (2011). Restricting microbial exposure in early life negates the immune benefits associated with gut colonization in environments of high microbial diversity. PLoS ONE.

[11] Hooper L.V., Littman D.R., Macpherson A.J. (2012). Interactions between the microbiota and the immune system. Science.

[12] Thompson C.L., Wang B., Holmes A.J. (2008). The immediate environment during postnatal development has long-term impact on gut community structure in pigs. ISME J..

[13] El-Aidy S., Hooiveld G., Tremaroli V., Bäckhed F., Kleerebezem M. (2013). The gut microbiota and mucosal homeostasis: Colonized at birth or at adulthood, does it matter?. Gut Microbes..

[14] Houghteling P.D., Walker W.A. (2015). Why is initial bacterial colonization of the intestine important to infants’ and children’s health?. J. Pediatr. Gastroenterol. Nutr..

[15] Hashemi A., Villa C.R., Comelli E.M. (2016). Probiotics in early life: A preventative and treatment approach. Food Funct..

[16] Gilliland S.E., Morelli L., Reid G. Health and nutritional properties of probiotics in food including powder milk with live lactic acid bacteria. Proceedings of the Joint FAO/WHO Expert Consultation on Evaluation of Health and Nutritional Properties of Probiotics in Food Including Powder Milk with Live Lactic Acid Bacteria.

[17] Zeyner A., Boldt E. (2006). Effects of a probiotic Enterococcus faecium strain supplemented from birth to weaning on diarrhoea patterns and performance of piglets. J. Anim. Physiol. Anim. Nutr..

[18] Liu H., Zhang J., Zhang S., Yang F., Thacker P.A., Zhang G., Qiao S., Ma X. (2014). Oral administration of lactobacillus fermentum I5007 favors intestinal development and alters the intestinal microbiota in formula-fed piglets. J. Agric. Food Chem..

[19] Strompfová V., Marciňáková M., Simonová M., Gancarčíková S., Jonecová Z., Sciranková Ľ., Koščová J., Buleca V., Čobanová K., Lauková A. (2006). Enterococcus faecium EK13—An enterocin A-producing strain with probiotic character and its effect in piglets. Anaerobe.

[20] Hou C., Liu H., Zhang J., Zhang S., Yang F., Zeng X., A Thacker P., Zhang G., Qiao S. (2015). Intestinal microbiota succession and immunomodulatory consequences after introduction of Lactobacillus reuteri I5007 in neonatal piglets. Loh G, editor. PLoS ONE.

[21] Lewis M.C., Merrifield C.A., Berger B., Cloarec O., Duncker S., Mercenier A., Nicholson J.K., Holmes E., Bailey M. (2017). Early intervention with Bifidobacterium lactis NCC2818 modulates the host-microbe interface independent of the sustained changes induced by the neonatal environment. Sci. Rep..

[22] Haupenthal L.A., Caramori Júnior J.G., Corrêa Gda S.S., Silva B.A.N. (2020). Oral supplementation of probiotics on the performance and gut histo-morphology of suckling piglets. Ciência Rural..

[23] Yang F., Wang A., Zeng X., Hou C., Liu H., Qiao S. (2015). Lactobacillus reuteri I5007 modulates tight junction protein expression in IPEC-J2 cells with LPS stimulation and in newborn piglets under normal conditions. BMC Microbiol..

[24] Luise D., Spinelli E., Correa F., Nicodemo A., Bosi P., Trevisi P. (2021). The effect of a single, early-life administration of a probiotic on piglet growth performance and faecal microbiota until weaning. Ital. J. Anim. Sci..

[25] Hansen L., Nielsen B., Boll E., Skjøt-Rasmussen L., Wellejus A., Jørgensen L., Lauridsen C., Canibe N. (2020). Functional in vitro screening of probiotic strains for inoculation of piglets as a prophylactic measure towards Enterotoxigenic Escherichia coli infection. J. Microbiol. Methods..

[26] Jørgensen C.B., Cirera S., Archibald A., Andersson L., Fredholm M., Edfors-Lilia I. (2010). Porcine Polymorphisms and Methods for Detecting Them. U.S. Patent.

[27] Hansen L.H.B., Lauridsen C., Nielsen B., Jørgensen L., Canibe N. (2022). Impact of early inoculation of probiotics to suckling piglets on postweaning diarrhoea–a challenge study with Enterotoxigenic *E. Coli* F18. Animal.

[28] Pedersen K.S., Toft N. (2011). Intra- and inter-observer agreement when using a descriptive classification scale for clinical assessment of faecal consistency in growing pigs. Prev. Vet. Med..

[29] Canibe N., Højberg O., Badsberg J.H., Jensen B.B. (2007). Effect of feeding fermented liquid feed and fermented grain on gastrointestinal ecology and growth performance in piglets. J. Anim. Sci..

[30] Skovgaard K., Cirera S., Vasby D., Podolska A., Breum S., Dürrwald R., Schlegel M., Heegaard P.M. (2013). Expression of innate immune genes, proteins and microRNAs in lung tissue of pigs infected experimentally with influenza virus (H1N2). Innate Immun..

[31] Brogaard L., Klitgaard K., Heegaard P.M.H., Hansen M.S., Jensen T.K., Skovgaard K. (2015). Concurrent host-pathogen gene expression in the lungs of pigs challenged with Actinobacillus pleuropneumoniae. BMC Genom..

[32] Edgar R.C. (2010). Search and clustering orders of magnitude faster than BLAST. Bioinformatics.

[33] Krych Ł., Kot W., Bendtsen K.M.B., Hansen A.K., Vogensen F.K., Nielsen D.S. (2018). Have you tried spermine? A rapid and cost-effective method to eliminate dextran sodium sulfate inhibition of PCR and RT-PCR. J. Microbiol. Methods.

[34] Edgar R. (2016). UNOISE2: Improved error-correction for Illumina 16S and ITS amplicon sequencing. bioRxiv.

[35] Edgar R. (2016). SINTAX: A simple non-Bayesian taxonomy classifier for 16S and ITS sequences. bioRxiv.

[36] R Core Team (2020). R: A language and environment for statistical computing. R Foundation for Statistical Computing [Internet]. https://www.r-project.org/.

[37] McMurdie P.J., Holmes S. (2013). phyloseq: An R package for reproducible interactive analysis and graphics of microbiome census data. PLoS ONE.

[38] Oksanen J., Simpson G.L., Blanchet F.G., Kindt R., Legendre P., Minchin P.R., O’Hara R., Solymos P., Stevens M.H.H., Szoecs E. (2018). R Package Version 2.1-41/r2867.2014; Vegan: Community Ecology Package. https://github.com/vegandevs/vegan.

[39] Bates D., Mächler M., Bolker B.M., Walker S.C. (2015). Fitting linear mixed-effects models using lme4. J. Stat. Softw..

[40] Wickham H. (2009). ggplot2.

[41] Kindt R., Coe R. (2005). Tree Diversity Analysis. A Manual and Software for Common Statistical Methods and Biodiversity Studies.

[42] Love M.I., Huber W., Anders S. (2014). Moderated estimation of fold change and dispersion for RNA-seq data with DESeq2. Genome Biol..

[43] Andersen K.S., Kirkegaard R.H., Karst S.M., Albertsen M. (2018). ampvis2: An R package to analyse and visualise 16S rRNA amplicon data. bioRxiv.

[44] Davis M.E., Brown D.C., Maxwell C.V., Rehberger T., Touchette K.J., Coalson J.A. Influence of Lactobacillus brevis 1E-1 on the gastrointestinal microflora, gut morphology, and pig performance pre-and post-weaning. Proceedings of the American Society of Animal Science Meeting.

[45] Gebert S., Davis E., Rehberger T., Maxwell C.V. (2011). Lactobacillus brevis strain 1E1 administered to piglets through milk supplementation prior to weaning maintains intestinal integrity after the weaning event. Benef. Microbes.

[46] Kiros T.G., Derakhshani H., Pinloche E., D’inca R., Marshall J., Auclair E., Khafipour E., Van Kessel A. (2018). Effect of live yeast Saccharomyces cerevisiae (Actisaf Sc 47) supplementation on the performance and hindgut microbiota composition of weanling pigs. Sci. Rep..

[47] Wang Q., Sun Q., Qi R., Wang J., Qiu X., Liu Z., Huang J. (2019). Effects of Lactobacillus plantarum on the intestinal morphology, intestinal barrier function and microbiota composition of suckling piglets. J. Anim. Physiol. Anim. Nutr..

[48] Liu H., Hou C., Wang G., Jia H., Yu H., Zeng X., A Thacker P., Zhang G., Qiao S. (2017). Lactobacillus reuteri I5007 modulates intestinal host defense peptide expression in the model of IPEC-J2 cells and neonatal piglets. Nutrients.

[49] Hu J., Chen L., Zheng W., Shi M., Liu L., Xie C., Wang X., Niu Y., Hou Q., Xu X. (2018). Lactobacillus frumenti facilitates intestinal epithelial barrier function maintenance in early-weaned piglets. Front. Microbiol..

[50] Siepert B., Reinhardt N., Kreuzer S., Bondzio A., Twardziok S., Brockmann G., Nöckler K., Szabó I., Janczyk P., Pieper R. (2014). Enterococcus faecium NCIMB 10415 supplementation affects intestinal immune-associated gene expression in post-weaning piglets. Vet. Immunol. Immunopathol..

[51] Del Mar Rigo-Adrover M., Franch À., Castell M., Pérez-Cano F.J. (2016). Preclinical immunomodulation by the probiotic Bifidobacterium breve M-16V in early life. PLoS ONE.

[52] Sayan H., Assavacheep P., Angkanaporn K., Assavacheep A. (2018). Effect of Lactobacillus salivarius on growth performance, diarrhea incidence, fecal bacterial population and intestinal morphology of suckling pigs challenged with F4+ enterotoxigenic *Escherichia coli*. Asian-Australas. J. Anim. Sci..

[53] Moeser A.J., Blikslager A.T. (2007). Mechanisms of porcine diarrheal disease. J. Am. Vet. Med. Assoc..

[54] Dubreuil J.D. (2017). Enterotoxigenic Escherichia coli targeting intestinal epithelial tight junctions: An effective way to alter the barrier integrity. Microb Pathog..

[55] Pomorska-Mól M., Kwit K., Markowska-Daniel I. (2012). Major acute phase proteins in pig serum from birth to slaughter. Bull. Vet. Inst. Pulawy..

[56] Heegaard P.M., Stockmarr A., Piñeiro M., Carpintero R., Lampreave F., Campbell F.M., Eckersall P.D., Toussaint M.J., Gruys E., Sorensen N.S. (2011). Optimal combinations of acute phase proteins for detecting infectious disease in pigs. Vet. Res..

[57] Wang Q., Meyer T.A., Boyce S.T., Wang J.J., Sun X., Tiao G., Fischer J.E., Hasselgren P.-O. (1998). Endotoxemia in mice stimulates production of complement C3 and serum amyloid A in mucosa of small intestine. Am. J. Physiol. Regul. Integr. Comp. Physiol..

[58] Liu C., Zhu Q., Chang J., Yin Q., Song A., Li Z., Wang E., Lu F. (2017). Effects of Lactobacillus casei and Enterococcus faecalis on growth performance, immune function and gut microbiota of suckling piglets. Arch. Anim. Nutr..

[59] Cooper T.A., Roberts M.P., Kattesh H.G., Kojima C.J. (2009). Effects of transport stress, sex, and weaning weight on post-weaning performance in pigs. Prof. Anim. Sci..

[60] Pluske J.R., Kim J.C., Black J.L. (2018). Manipulating the immune system for pigs to optimise performance. Anim. Prod. Sci..

[61] Cox L.M., Yamanishi S., Sohn J., Alekseyenko A.V., Leung J.M., Cho I., Kim S.G., Li H., Gao Z., Mahana D. (2014). Altering the intestinal microbiota during a critical developmental window has lasting metabolic consequences. Cell.

[62] Benis N., Schokker D., Suarez-Diez M., Martins dos Santos V.A.P., Smidt H., Smits M.A. (2015). Network analysis of temporal functionalities of the gut induced by perturbations in new-born piglets. BMC Genom..

[63] De la Cuesta-Zuluaga J., Mueller N.T., Álvarez-Quintero R., Velásquez-Mejía E.P., Sierra J.A., Corrales-Agudelo V., Carmona J.A., Abad J.M., Escobar J.S. (2019). Higher fecal short-chain fatty acid levels are associated with gut microbiome dysbiosis, obesity, hypertension and cardiometabolic disease risk factors. Nutrients.

[64] Kelly C.J., Zheng L., Campbell E.L., Saeedi B., Scholz C.C., Bayless A.J., Wilson K.E., Glover L.E., Kominsky D.J., Magnuson A. (2015). Crosstalk between microbiota-derived short-chain fatty acids and intestinal epithelial HIF augments tissue barrier function. Cell Host Microbe.

[65] Grillo M.A., Colombatto S. (2003). Metabolism and function in animal tissues of agmatine, a biogenic amine formed from arginine. Amino Acids.

[66] Porter P., Kenworthy R. (1969). A study of intestinal and urinary amines in pigs in relation to weaning. Res. Vet. Sci..

[67] Bian G., Ma S., Zhu Z., Su Y., Zoetendal E.G., Mackie R., Liu J., Mu C., Huang R., Smidt H. (2016). Age, introduction of solid feed and weaning are more important determinants of gut bacterial succession in piglets than breed and nursing mother as revealed by a reciprocal cross-fostering model. Environ. Microbiol..

[68] Hurley W.L., Farmer C. (2015). Composition of sow colostrum and milk. The Gestating and Lactating Sow.

