# Supplementary material for: Early Inoculation of a Multi-Species Probiotic in Piglets–Impacts on the Gut Microbiome and Immune Responses
Source: Microorganisms. 2025 May 31;13(6):1292. doi: 10.3390/microorganisms13061292 (PMC12194841; doi:10.3390/microorganisms13061292)
Supplement: Supplementary file 1 [file microorganisms-13-01292-s001.zip › microorganisms-3632711-supplementary.pdf]

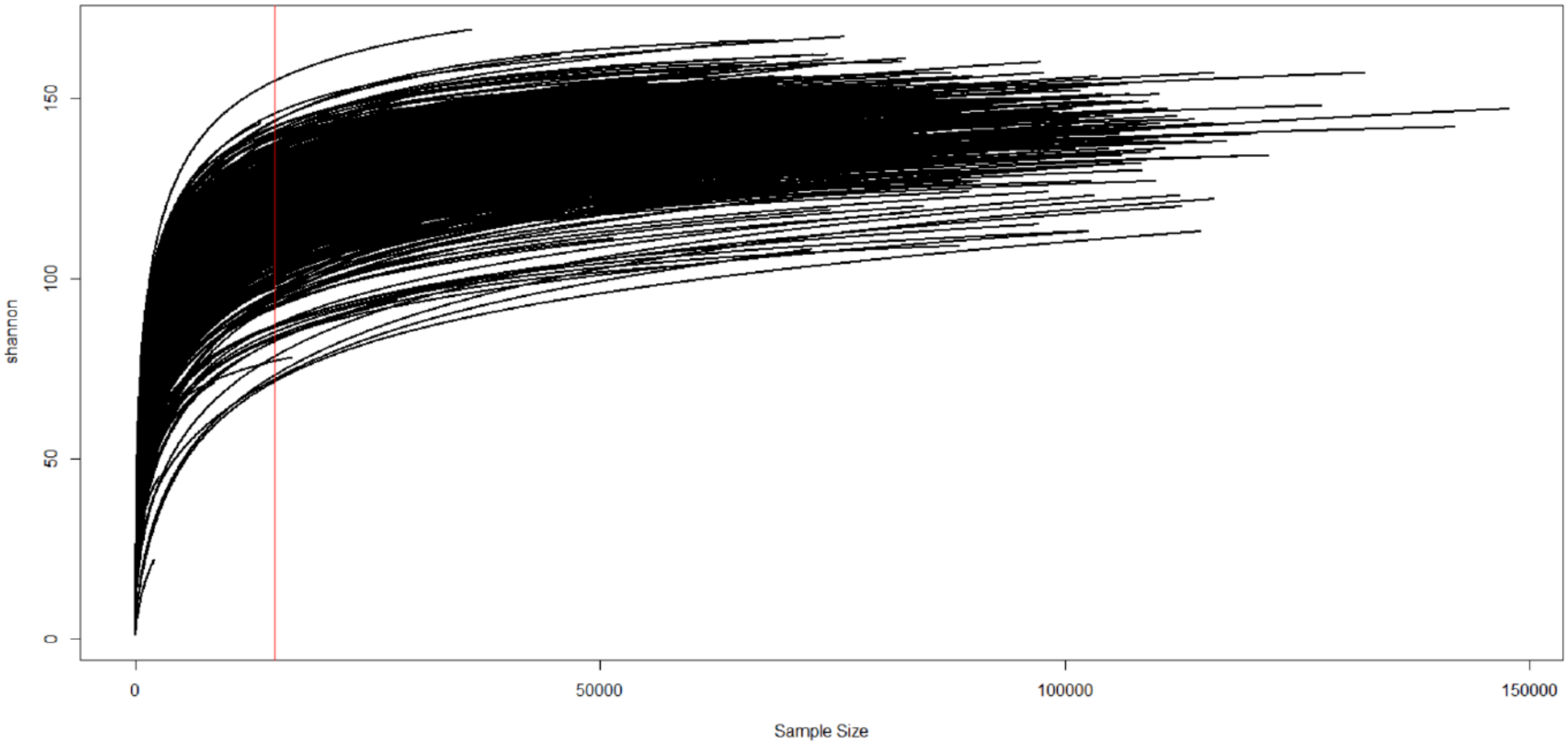


**Figure S1.** Rarefaction curve of all samples. Shannon is displayed. The red line at 15 000 reads per sample indicates the read depth chosen for rarefication.

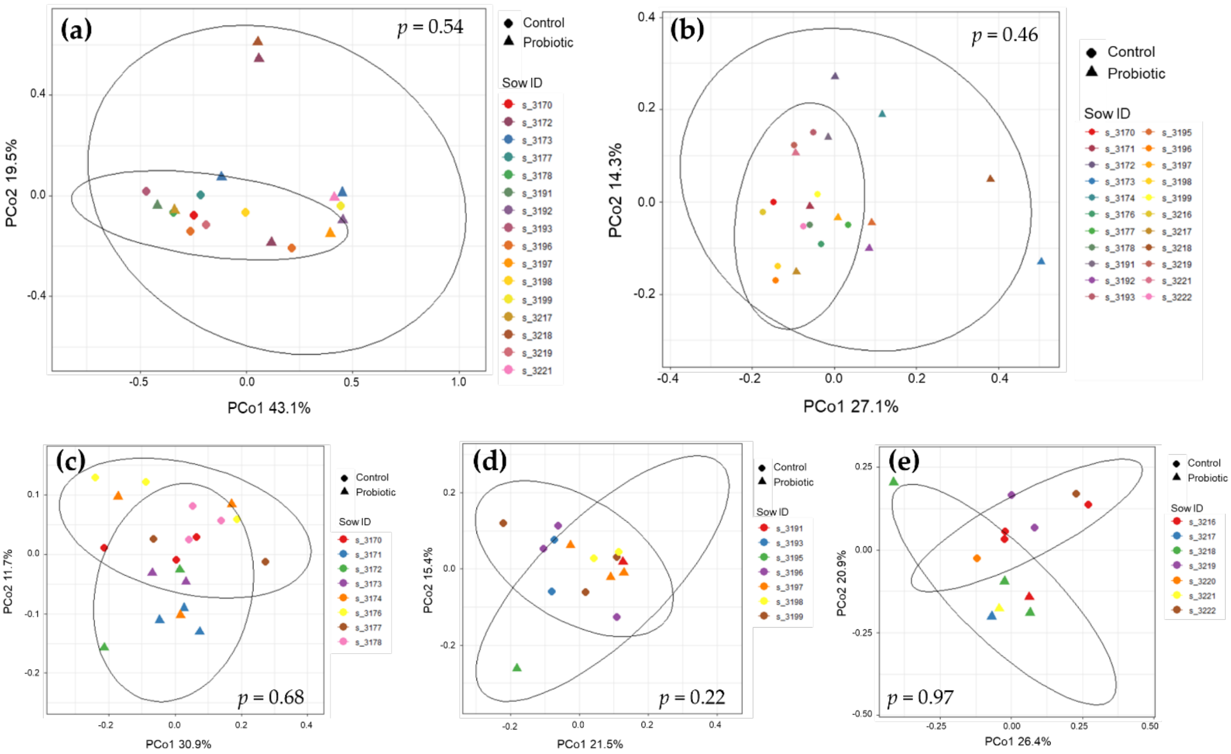


**Figure S2.** Principal coordinates analysis (PCoA) of Bray-Curtis dissimilarity between the Control and Probiotic group on day 35. Bray-Curtis distance metrics were used to compare the composition of the microbiota between the two treatment groups in (**a**) small intestinal mucosa, (**b**) digesta of the proximal colon, and feces in round (**c**) 1, (**d**) 2, and (**e**) 3. Nested permutational multivariate analysis of variance (PERMANOVA) on Bray-Curtis distance metrics with sow ID nested with treatment group was carried out using 999 permutations to test for significance of clustering pattern. P-values for the effect of sow are illustrated. $p < 0.05$ was considered significant whereas $p < 0.10$ was considered as a statistical tendency.

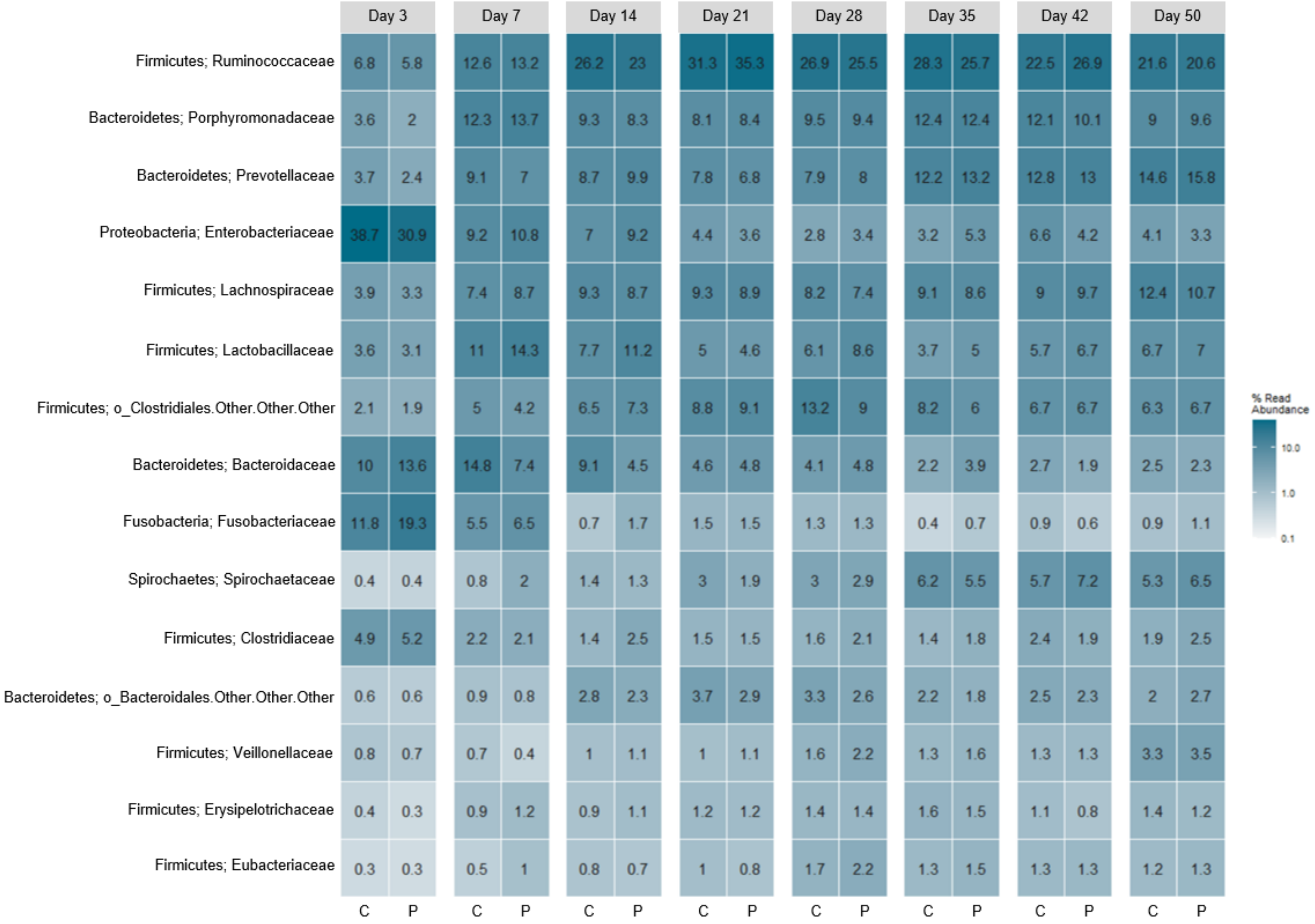


**Figure S3.** Heatmap of fecal samples collected at day 3, 7, 14, 21, 28, 35, 42 and 50 from pigs administered placebo (C) or probiotics (P) during suckling (d1–28). The heatmap shows the relative abundance (%) of the 15 most abundant families in feces. Colors represent relative abundance. Number of samples: Control d3 = 21, d7 = 33, d14 = 29, d21 = 29, d28 = 28, d35 = 28, d42 = 28, d50 = 27. Probiotic d3 = 29, d7 = 32, d14 = 25, d21 = 24, d28 = 21, d35 = 21, d42 = 21, d50 = 21.

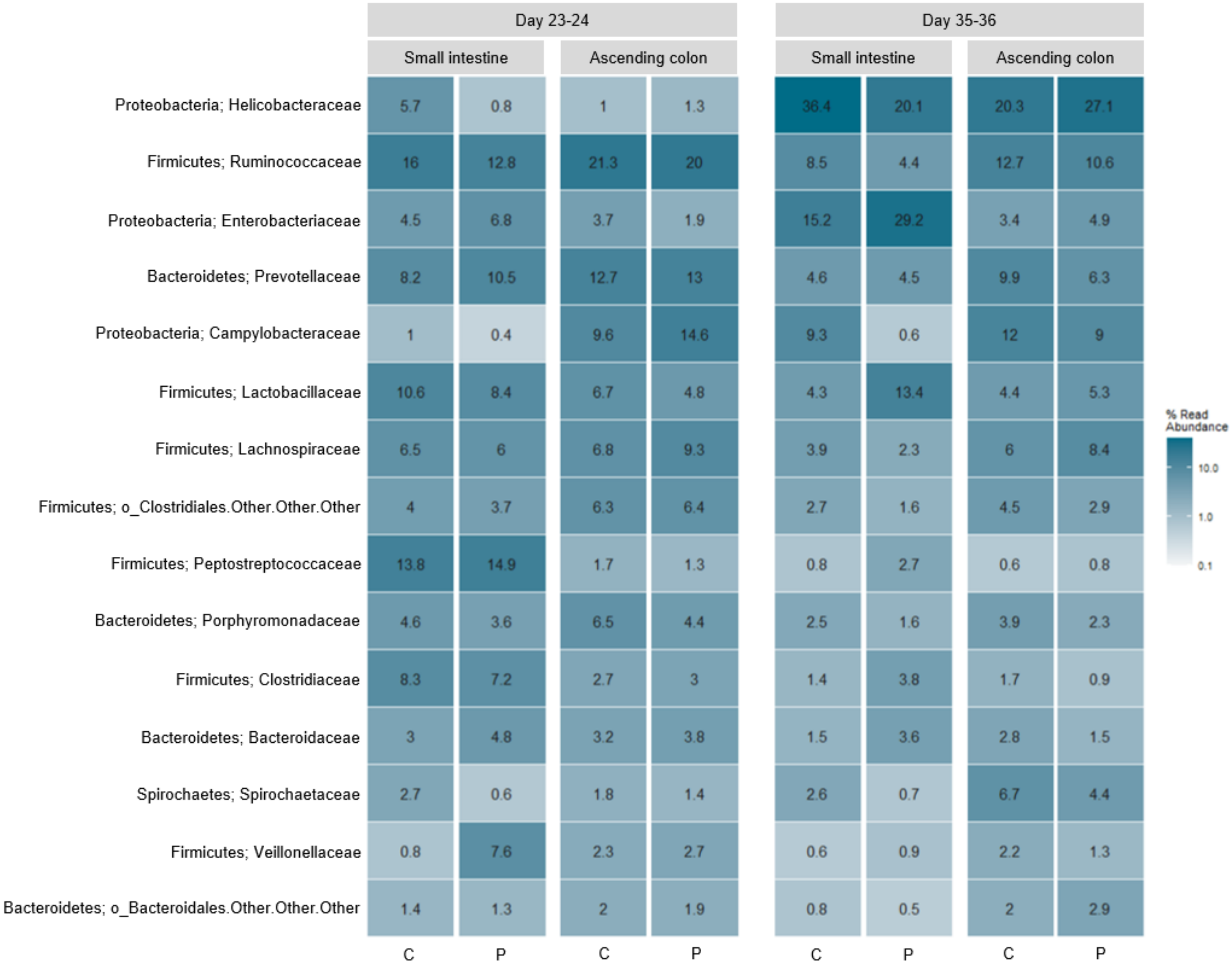


**Figure S4.** Heatmap of mucosal samples collected from the small intestine and ascending colon (CO1) at day 23–24 (pre-weaning) and day 35–36 (post-weaning). Pigs were administered placebo (C) or probiotics (P) during suckling (d1–28). The heatmap shows the relative abundance (%) of the 15 most abundant families in mucosa. Colors represent relative abundance. Number of samples: Control d23–24 = 20, d35–36 = 20. Probiotic d23–24 = 14, d35–36 = 20.

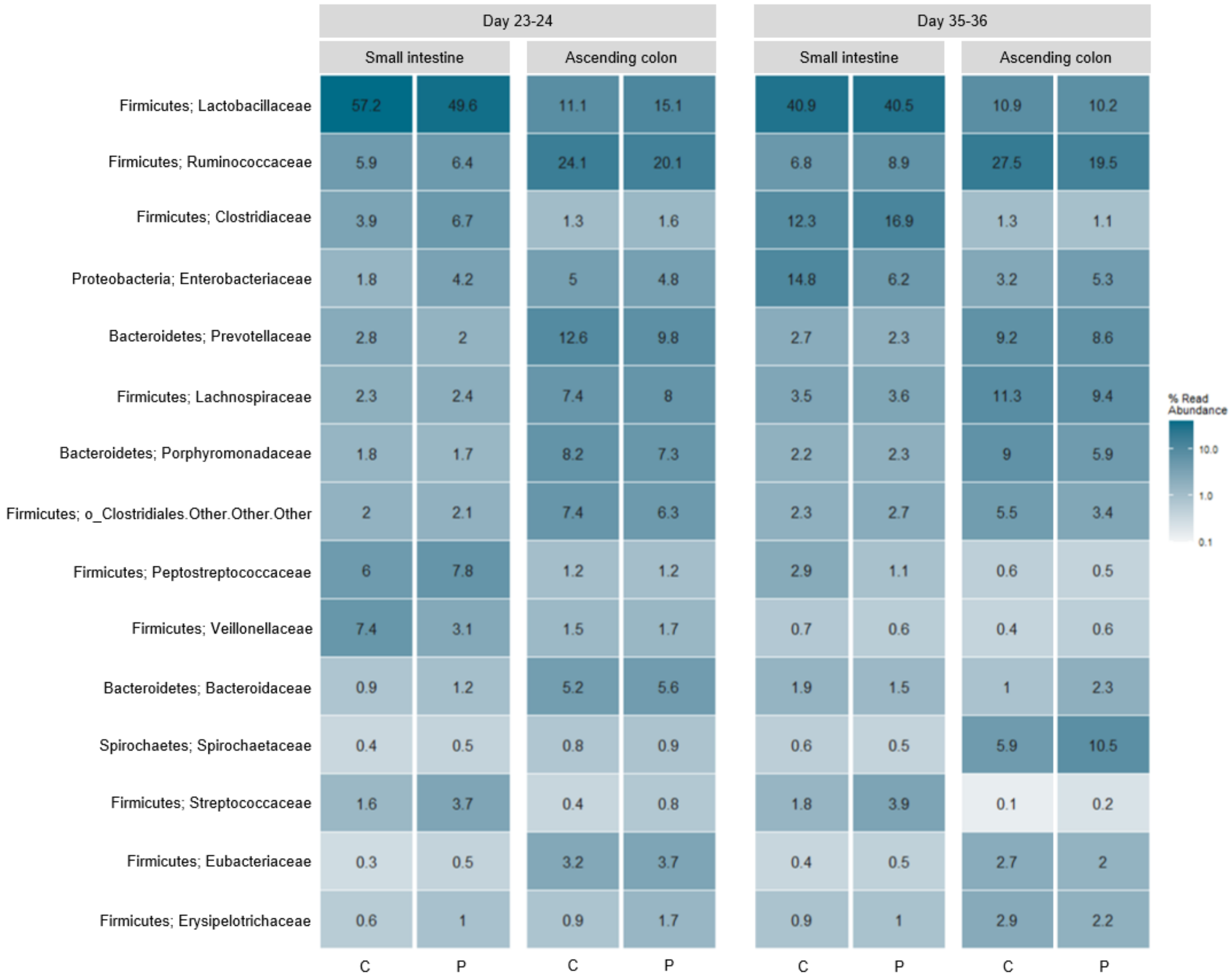


**Figure S5.** Heatmap of digesta samples collected from the small intestine and ascending colon (CO1) at day 23–24 (pre-weaning) and day 35–36 (post-weaning). Pigs were administered placebo (C) or probiotics (P) during suckling (d1–28). The heatmap shows the relative abundance (%) of the 15 most abundant families in digesta. Colors represent relative abundance. Number of samples: Control d23–24 = 33, d35–36 = 26. Probiotic d23–24 = 32, d35–36 = 26.

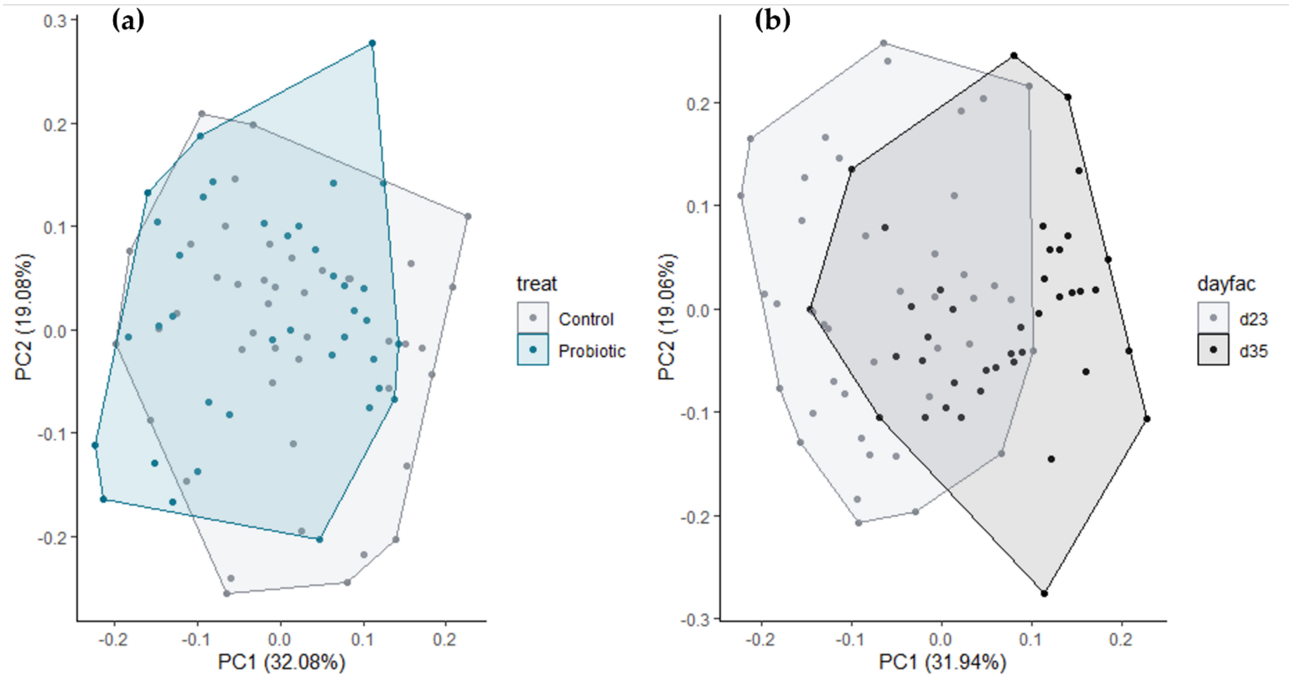


**Figure S6.** Principle component analysis (PCA) plot on $Log_2$ transformed gene expression data discriminated between samples belonging to a) the two different treatment groups (Control and Probiotic), or b) the two different time points (day 23–24 or day 35–36). Number of samples: Control d23–24 = 22, Control d35–36 = 22. Probiotic d23–24 = 20, Probiotic d35–36 = 18.

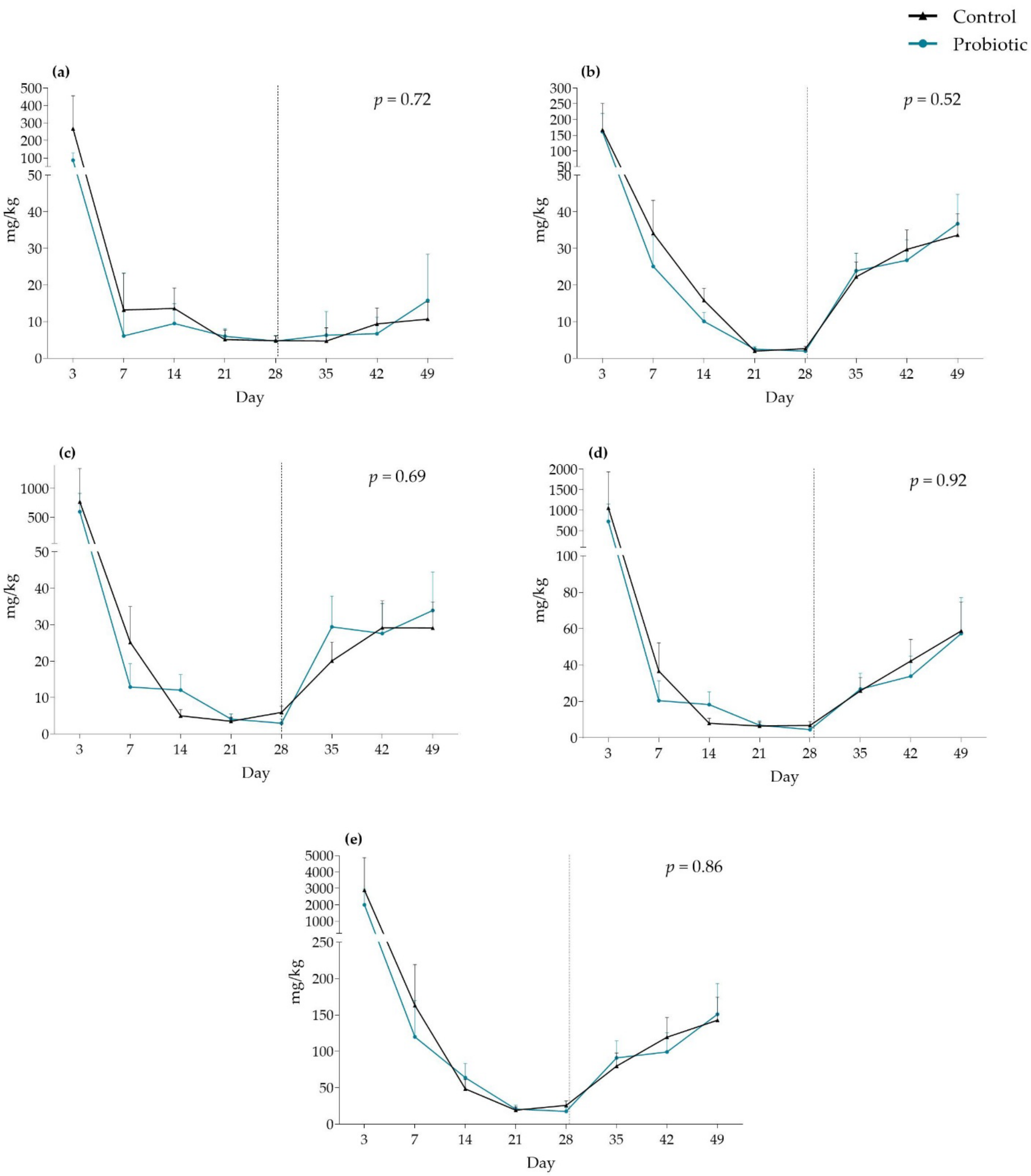


**Figure S7.** Effect of early probiotic inoculation on the biogenic amines **(a)** tyramine, **(b)** agmatine, **(c)** putrescine, **(d)** cadaverine, and **(e)** the total concentration of biogenic amines in feces (mg/kg). The dotted line illustrates the day of weaning. Values are presented as least square means and SE, and the *P*-value for effect of probiotic treatment is stated. Number of samples: Control d3 = 3, d7 = 12, d14 = 21, d21 = 26, d28 = 27, d35 = 27, d42 = 27, d50 = 28. Probiotic d3 = 6, d7 = 8, d14 = 15, d21 = 22, d28 = 18, d35 = 21, d42 = 20, d50 = 18.

**Table S1.** mRNA primer sequences and amplicon length (F: Forward, R: Reverse).

| Gene Symbol | Gene Name | Sequence | Amplicon Length |
|---|---|---|---|
| GAPDH | Glyceraldehyde-3-phosphate dehydrogenase | F: ACCCAGAAGACTGTGGATGG<br>R: AAGCAGGGATGATGTTCTGG | 79 |
| TBP | TATA box binding protein | F: ACGTTCGGTTTAGGTTGCAG<br>R: CAGGAACGCTCTGGAGTTCT | 96 |
| B2M | Beta-2-Microglobulin | F: TGAAGCACGTGACTCTCGAT<br>R: CTCTGTGATGCCGGTTAGTG | 70 |
| ZO1 | Tight Junction Protein 1 (ZO1) | F: ATGACTCCTGACGGTTGGTC<br>R: TGCCAGGTTTTAGGATCACC | 71 |
| OCLN | Occludin | F: GACGAGCTGGAGGAAGACTG<br>R: GTACTCCTGCAGGCCACTGT | 102 |
| MUC2 | Mucin 2 | F: GCACGTCTGCAACAAGGAC<br>R: CAAAGCCCTCCAGGCAGT | 125 |
| MUC1 | Mucin 1 | F: GGATTTCTGAATTGTTTTTGCAG<br>R: ACTGTCTTGGAAGGCCAGAA | 116 |
| SLC5A1 | Solute Carrier Family 5 (Sodium/Glucose Cotransporter), Member 1 | F: CTGCAAGAGAGTCAATGAGGAG<br>R: CCGGTTCCATAGGCAAACT | 99 |
| SLC5A8 | Solute carrier family 5 member 8 | F: TGGGACAAATTGGATGACAA<br>R: CCATCAGTGGAGTCCTTTCAA | 86 |
| SLC2A2 | Solute Carrier Family 2 (Facilitated Glucose Transporter), Member 2 | F: CATGTCAGTGGGACTTGTGC<br>R: TGGCCCAATTTCAAAGAAAC | 100 |
| SLC16A1 | Solute Carrier Family 16 Member 1 | F: CCGACTTCTGGCAAAAGAAC<br>R: GGCTTCTCAGCAGCGTCTAT | 90 |
| PPIA | peptidylprolyl isomerase A (cyclophilin A) | F: CAAGACTGAGTGGTTGGATGG<br>R: TGTCCACAGTCAGCAATGGT | 138 |
| IL1RAP | Interleukin 1 Receptor Accessory Protein | F: GCATCACCTCCCCAAATCTA<br>R: GTAGCTCCTCTCCCGGTTCT | 70 |
| SAA | Serum Amyloid A | F: TGGAGAGCCTACTCGGACAT<br>R: CCTTTGGGCAGCATCATAGT | 90 |
| TNFa | Tumor Necrosis Factor alpha | F: CCCCCAGAAGGAAGAGTTTC<br>R: CGGGCTTATCTGAGGTTTGA | 92 |
| IL–23p19 | Interleukin 23 p13 Subunit | F: CAACAGTCAGTCCTGCTTGC<br>R: GCTCCCCTGTGAAAATGTCT | 86 |
| IL–18 | Interleukin 18 | F: CAATTGCATCAGCTTTGTGG<br>R: TCCAGGTCCTCATCGTTTTC | 78 |
| IL–17 | Interleukin 17F | F: AATCAGGGAGTTCCCCTCTC | 75 |

| | | | |
|---|---|---|---|
| | | R: GTCCCGGGTGATGTTGTAAT | |
| IL–12p40 | Interleukin 12 p40 | F: GACCAGAAAGAGCCCAAAAAC | 70 |
| | | R: AGGTGAAACGTCCGGAGTAA | |
| IL–8 | Interleukin 8 | F: AAGAGAACTGAGAAGCAACAACA | 99 |
| | | R: TTGTGTTGGCATCTTTACTGAGA | |
| IL–1B | Interleukin 1, Beta | F: TCTCTCACCCCTTCTCCTCA | 60 |
| | | R: GACCCTAGTGTGCCATGGTT | |
| IL–10 | Interleukin 10 | F: TACAACAGGGGCTTGCTCTT | 110 |
| | | R: GCCAGGAAGATCAGGCAATA | |

**Table S2.** Effect of early inoculation of probiotics on DL-lactic acid, short-chain fatty acids and branched fatty acids in small intestinal (SI) and colonic (CO) intestinal content on day 23–24 pre-weaning [1,2].

| | Control | Probiotic | SEM | * | *P*-value [3] | | |
|---|---|---|---|---|---|---|---|
| | | | | | Treat | Segment | Treat x Segment |
| DL–Lactic acid (mmol/kg) | | | | | 0.16 | <0.001 | 0.31 |
| Proximal SI | 8.7 | 6.8 | 1.5 | a | | | |
| Distal SI | 4.7 | 2.9 | 0.8 | b | | | |
| Branched fatty acid [4] (mmol/kg) | | | | | 0.60 | <0.001 | 0.48 |
| Proximal CO | 3.7 | 3.7 | 0.4 | a | | | |
| Mid CO | 2.1 | 1.9 | 0.3 | b | | | |
| Distal CO | 2.3 | 1.9 | 0.3 | b | | | |
| Total SCFA [5] (mmol/kg) | | | | | 0.67 | <0.001 | 0.61 |
| Proximal SI | 1.1 | 0.8 | 0.3 | a | | | |
| Distal SI | 9.2 | 11.8 | 1.2 | a | | | |
| Proximal CO | 65.3 | 72.4 | 5.8 | b | | | |
| Mid CO | 35.5 | 37.3 | 5.4 | c | | | |
| Distal CO | 33.3 | 30.0 | 3.2 | c | | | |
| Acetic acid [6] (%) | | | | | 0.99 | <0.001 | 0.20 |
| Proximal SI | 74.2 | 74.1 | 1.6 | a | | | |
| Distal SI | 77.4 | 71.0 | 2.4 | b | | | |
| Proximal CO | 65.0 | 66.6 | 1.8 | c | | | |
| Mid CO | 66.9 | 67.2 | 2.0 | c | | | |
| Distal CO | 69.5 | 68.8 | 2.2 | bc | | | |
| Formic acid [6] (%) | | | | | 0.20 | | |
| Distal SI | 16.9 | 21.3 | 2.2 | | | | |
| Butyric acid [6] (%) | | | | | 1.00 | 0.04 | 0.17 |
| Proximal CO | 8.8 | 8.3 | 0.7 | a | | | |
| Mid CO | 9.3 | 9.2 | 0.8 | ab | | | |
| Distal CO | 8.1 | 8.8 | 0.7 | ac | | | |
| | | | | | 0.81 | <0.001 | 0.91 |

| | | | | | | | |
|---|---|---|---|---|---|---|---|
| Propionic acid [6] (%) | | | | | | | |
| Proximal SI | 1.0 | 1.5 | 0.8 | a | | | |
| Distal SI | 4.3 | 4.8 | 2.0 | b | | | |
| Proximal CO | 22.2 | 20.9 | 1.1 | c | | | |
| Mid CO | 19.6 | 19.3 | 1.0 | c | | | |
| Distal CO | 18.0 | 18.2 | 0.9 | c | | | |
| Valeric acid [6] (%) | | | | | 0.92 | 0.81 | 0.62 |
| Proximal CO | 3.5 | 3.5 | 0.3 | | | | |
| Mid CO | 3.6 | 3.5 | 0.3 | | | | |
| Distal CO | 3.5 | 3.7 | 0.3 | | | | |

[1] Values are presented as least square means and SEM

[2] Number of samples: Control $n$ = 11. Probiotic $n$ = 11

[3] Treat = Treatment group, Segment = Intestinal segment, Treat x Segment = interaction between treatment and intestinal segment.

[4] Branched fatty acid: Isovaleric and isobutyric acid.

[5] SCFA: Formic, acetic, propionic, butyric, and valeric acid.

[6] Percent of the specific acid of total SCFA.

* Different lowercase letters indicate statistical significance ($p < 0.05$) between intestinal segments.

**Table S3.** Effect of early inoculation of probiotics on DL-lactic acid, short-chain fatty acids and branched fatty acids in small intestinal (SI) and colonic (CO) intestinal content on day 35–36 post-weaning [1,2].

| | Control | Probiotic | SEM | * | *P*-value [3] | | |
|---|---|---|---|---|---|---|---|
| | | | | | **Treat** | **Segment** | **Treat x Segment** |
| DL–Lactic acid (mmol/kg) | | | | | 0.76 | <0.001 | 0.16 |
| Proximal SI | 3.5 | 3.8 | 1.1 | a | | | |
| Distal SI | 6.4 | 5.1 | 1.7 | b | | | |
| Branched fatty acid [4] (mmol/kg) | | | | | 0.13 | <0.001 | 0.12 |
| Proximal CO | 2.0 | 1.4 | 0.2 | a | | | |
| Mid CO | 2.6 | 2.1 | 0.3 | b | | | |
| Distal CO | 2.6 | 2.5 | 0.3 | b | | | |
| Total SCFA [5] (mmol/kg) | | | | | 0.48 | <0.001 | 0.53 |
| Proximal SI | 0.9 | 0.9 | 0.1 | a | | | |
| Distal SI | 6.1 | 7.8 | 1.4 | a | | | |
| Proximal CO | 99.9 | 91.7 | 7.9 | b | | | |
| Mid CO | 87.0 | 82.5 | 6.0 | c | | | |
| Distal CO | 78.2 | 69.5 | 4.2 | d | | | |
| Acetic acid [6] (%) | | | | | 0.53 | <0.001 | 1.00 |
| Proximal SI | 94.7 | 96.7 | 2.8 | a | | | |
| Distal SI | 61.3 | 64.1 | 5.3 | b | | | |
| Proximal CO | 65.1 | 66.1 | | b | | | |
| Mid CO | 64.8 | 66.7 | 2.3 | b | | | |

| | | | | | | | |
|---|---|---|---|---|---|---|---|
| Distal CO | 63.9 | 65.3 | 1.8 | b | | | |
| Formic acid [6] (%) | | | | | 0.99 | | |
| Distal SI | 35.7 | 35.1 | 5.3 | | | | |
| Butyric acid [6] (%) | | | | | 0.34 | 0.001 | 0.30 |
| Proximal CO | 8.5 | 6.9 | 1.1 | a | | | |
| Mid CO | 8.9 | 7.0 | 1.1 | a | | | |
| Distal CO | 9.2 | 8.0 | 1.2 | b | | | |
| Propionic acid [6] (%) | | | | | 0.41 | <0.001 | 0.93 |
| Proximal SI | 1.2 | 0.7 | 0.6 | a | | | |
| Distal SI | 0.3 | 0.1 | 0.2 | a | | | |
| Proximal CO | 23.7 | 23.6 | 0.9 | b | | | |
| Mid CO | 23.5 | 22.8 | 0.9 | b | | | |
| Distal CO | 24.2 | 23.6 | 0.9 | b | | | |
| Valeric acid [6] (%) | | | | | 0.06 | 0.01 | 0.01 |
| Proximal CO | 2.2 | 1.5 | 0.2 | a | | | |
| Mid CO | 2.3 | 1.8 | 0.2 | ab | | | |
| Distal CO | 2.2 | 2.1 | 0.2 | b | | | |

[1] Values are presented as least square means and SEM

[2] Number of samples: Control $n$ = 11. Probiotic $n$ = 11

[3] Treat = Treatment group, Segment = Intestinal segment, Treat x Segment = interaction between treatment and intestinal segment.

[4] Branched fatty acid: Isovaleric and isobutyric acid.

[5] SCFA: Formic, acetic, propionic, butyric, and valeric acid.

[6] Percent of the specific acid of total SCFA.

* Different lowercase letters indicate statistical significance ($p < 0.05$) between intestinal segments.

**Table S4.** Effect of early inoculation of probiotics on biogenic amines in small intestinal (SI) and colonic (CO) intestinal content on day 23–24 pre-weaning (mg/kg sample) [1,2].

| | **Control** | **Probiotic** | **SEM** | ***** | ***P*-value[3]** | | |
|---|---|---|---|---|---|---|---|
| | | | | | **Treat** | **Segment** | **Treat x Segment** |
| Agmatine | | | | | 0.10 | <0.001 | 0.47 |
| Distal SI | 63.8 | 65.3 | 7.6 | a | | | |
| Proximal CO | 14.4 | 20.0 | 2.0 | b | | | |
| Mid CO | 11.1 | 14.2 | 1.5 | c | | | |
| Distal CO | 10.9 | 11.8 | 1.3 | c | | | |
| Putrescine | | | | | 0.80 | <0.001 | 0.99 |
| Distal SI | 35.3 | 40.4 | 4.5 | a | | | |
| Proximal CO | 53.5 | 54.6 | 12.8 | a | | | |
| Mid CO | 36.1 | 38.4 | 8.9 | a | | | |
| Distal CO | 21.3 | 22.7 | 5.3 | b | | | |
| Cadaverine | | | | | 0.87 | 0.008 | 0.99 |
| Distal SI | 21.7 | 23.5 | 9.1 | a | | | |

| | | | | | | | |
|---|---|---|---|---|---|---|---|
| Proximal CO | 45.9 | 51.7 | 19.3 | ab | | | |
| Mid CO | 29.3 | 34.4 | 12.7 | a | | | |
| Distal CO | 24.4 | 24.4 | 9.8 | ac | | | |
| Tyramine | | | | | 0.26 | 0.02 | 0.04 |
| Distal SI | 9.9[a] | 2.4[b] | 2.4 | a | | | |
| Proximal CO | 11.2 | 7.0 | 3.4 | ab | | | |
| Mid CO | 6.7 | 5.0 | 2.3 | a | | | |
| Distal CO | 5.6 | 4.2 | 2.0 | ac | | | |
| Sum | | | | | 0.88 | <0.001 | 0.98 |
| Distal SI | 165.7 | 179.6 | 37.0 | a | | | |
| Proximal CO | 144.8 | 147.5 | 31.3 | a | | | |
| Mid CO | 91.1 | 99.9 | 20.5 | b | | | |
| Distal CO | 68.6 | 70.6 | 14.9 | c | | | |

[1] Values are presented as least square means and SEM. Different lowercase letters next to means indicate statistical significance ($P < 0.05$) between treatment groups in a specific intestinal segment

[2] Number of samples: Control $n = 11$. Probiotic $n = 11$

[3] Treat = Treatment group, Segment = Intestinal segment, Treat x Segment = interaction between treatment and intestinal segment.

* Different lowercase letters indicate statistical significance ($p < 0.05$) between intestinal segments.

**Table S5.** Effect of early inoculation of probiotics on biogenic amines in small intestinal (SI) and colonic (CO) intestinal content on day 35–36 post-weaning (mg/kg sample)[1,2].

| | **Control** | **Probiotic** | **SEM** | * | ***P*-value[3]** | | |
|---|---|---|---|---|---|---|---|
| | | | | | **Treat** | **Segment** | **Treat x Segment** |
| Agmatine | | | | | 0.74 | <0.001 | 0.04 |
| Distal SI | 33.9 | 28.3 | 2.6 | a | | | |
| Proximal CO | 17.8 | 16.0 | 1.4 | b | | | |
| Mid CO | 18.3[a] | 20.7[b] | 1.6 | b | | | |
| Distal CO | 18.4[a] | 22.5[b] | 1.7 | b | | | |
| Putrescine | | | | | 0.92 | <0.001 | 0.96 |
| Distal SI | 2.4 | 2.4 | 0.6 | a | | | |
| Proximal CO | 49.2 | 43.5 | 8.7 | b | | | |
| Mid CO | 52.3 | 52.7 | 9.8 | b | | | |
| Distal CO | 54.2 | 57.5 | 10.4 | b | | | |
| Cadaverine | | | | | 0.09 | <0.001 | 0.39 |
| Distal SI | 2.7 | 0.6 | 0.7 | a | | | |
| Proximal CO | 57.5 | 32.8 | 12.1 | b | | | |
| Mid CO | 65.0 | 46.9 | 14.9 | b | | | |
| Distal CO | 60.0 | 52.5 | 14.9 | b | | | |
| Tyramine | | | | | 0.67 | <0.001 | 0.16 |
| Distal SI | 0.4 | 0.4 | 0.4 | a | | | |

| | | | | | | | |
|---|---|---|---|---|---|---|---|
| Proximal CO | 1.4 | 0.9 | 0.6 | ab | | | |
| Mid CO | 1.2 | 1.8 | 0.7 | b | | | |
| Distal CO | 1.1 | 2.6 | 0.8 | b | | | |
| Sum | | | | | 0.43 | <0.001 | 0.51 |
| Distal SI | 43.1 | 36.2 | 5.5 | a | | | |
| Proximal CO | 134.9 | 102.9 | 16.5 | b | | | |
| Mid CO | 149.8 | 132.9 | 19.7 | b | | | |
| Distal CO | 143.9 | 150.4 | 20.5 | b | | | |

[1] Values are presented as least square means and SEM. Different lowercase letters next to means indicate statistical significance ($P < 0.05$) between treatment groups in a specific intestinal segment

[2] Number of samples: Control $n = 11$. Probiotic $n = 11$

[3] Treat = Treatment group, Segment = Intestinal segment, Treat x Segment = interaction between treatment and intestinal segment.

* Different lowercase letters indicate statistical significance ($p < 0.05$) between intestinal segments.

**Table S6.** Effect of early inoculation of probiotics on pH in content from the stomach, small intestine (SI), caecum, and colon (CO) on day 23–24 pre-weaning[1,2].

| | Control | Probiotic | SEM | * | *P*-value[3] | | |
|---|---|---|---|---|---|---|---|
| | | | | | **Treat** | **Segment** | **Treat x Segment** |
| | | | | | 0.61 | <0.001 | 0.96 |
| Stomach | 3.6 | 3.6 | 0.3 | a | | | |
| Proximal SI | 6.2 | 6.1 | 0.1 | b | | | |
| Distal SI | 7.0 | 7.1 | 0.04 | c | | | |
| Caecum | 6.4 | 6.4 | 0.1 | bc | | | |
| Proximal CO | 6.7 | 6.6 | 0.04 | c | | | |
| Mid CO | 6.6 | 6.6 | 0.1 | c | | | |
| Distal CO | 6.6 | 6.5 | 0.1 | c | | | |

[1] Values are presented as least square means and SEM

[2] Number of samples: Control $n = 23$. Probiotic $n = 20$

[3] Treat = Treatment group, Segment = Intestinal segment, Treat x Segment = interaction between treatment and intestinal segment.

* Different lowercase letters indicate statistical significance ($p < 0.05$) between intestinal segments.

**Table S7.** Effect of early inoculation of probiotics on pH in content from the stomach, small intestine (SI), caecum, and colon (CO) on day 35–36 post-weaning[1,2.]

| | Control | Probiotic | SEM | * | *P*-value[3] | | |
|---|---|---|---|---|---|---|---|
| | | | | | Treat | Segment | Treat x Segment |
| | | | | | 0.20 | <0.001 | 0.005 |
| Stomach | 2.9[a] | 3.7[b] | 0.2 | a | | | |
| Proximal SI | 6.1 | 6.1 | 0.1 | b | | | |
| Distal SI | 6.8 | 7.0 | 0.1 | c | | | |
| Caecum | 6.1 | 6.1 | 0.1 | b | | | |
| Proximal CO | 6.4 | 6.4 | 0.1 | bc | | | |
| Mid CO | 6.7 | 6.7 | 0.1 | cd | | | |
| Distal CO | 6.8 | 6.9 | 0.1 | cd | | | |

[1] Values are presented as least square means and SEM. Different lowercase letters next to means indicate statistical significance ($p < 0.05$) between treatment groups in a specific intestinal segment.

[2] Number of samples: Control $n = 23$. Probiotic $n = 18$

[3] Treat = Treatment group, Segment = Intestinal segment, Treat x Segment = interaction between treatment and intestinal segment.

* Different lowercase letters indicate statistical significance ($p < 0.05$) between intestinal segments.

**Table S8.** Effect of early inoculation of probiotics on dry matter percentage (DM%) in content from the stomach, small intestine (SI), caecum, and colon (CO) on day 23–24 pre-weaning[1,2.]

| | Control | Probiotic | SEM | * | *P*-value[3] | | |
|---|---|---|---|---|---|---|---|
| | | | | | Treat | Segment | Treat x Segment |
| | | | | | 0.21 | <0.001 | 0.73 |
| Stomach | 24.4 | 23.4 | 1.7 | a | | | |
| Proximal SI | 12.9 | 10.7 | 0.9 | b | | | |
| Distal SI | 13.8 | 13.3 | 1.1 | b | | | |
| Caecum | 14.1 | 13.5 | 1.0 | b | | | |
| Proximal CO | 21.4 | 18.9 | 1.5 | ac | | | |
| Mid CO | 22.6 | 20.5 | 1.6 | ac | | | |
| Distal CO | 27.5 | 28.3 | 2.3 | ad | | | |

[1] Values are presented as least square means and SEM

[2] Number of samples: Control $n = 23$. Probiotic $n = 20$

[3] Treat = Treatment group, Segment = Intestinal segment, Treat x Segment = interaction between treatment and intestinal segment.

* Different lowercase letters indicate statistical significance ($p < 0.05$) between intestinal segments.

**Table S9.** Effect of early inoculation of probiotics on dry matter percentage in content from the stomach, small intestine (SI), caecum, and colon (CO) on day 35–36 post-weaning (DM%) [1,2].

| | **Control** | **Probiotic** | **SEM** | ***** | ***P*-value[3]** | | |
|---|---|---|---|---|---|---|---|
| | | | | | **Treat** | **Segment** | **Treat x Segment** |
| | | | | | 0.34 | <0.001 | 0.82 |
| Stomach | 18.4 | 18.2 | 2.2 | a | | | |
| Proximal SI | 11.0 | 9.3 | 1.2 | b | | | |
| Distal SI | 6.9 | 5.7 | 0.8 | c | | | |
| Caecum | 6.8 | 6.7 | 0.8 | c | | | |
| Proximal CO | 11.4 | 9.3 | 1.3 | d | | | |
| Mid CO | 15.5 | 14.4 | 1.8 | ae | | | |
| Distal CO | 17.5 | 17.9 | 2.1 | ae | | | |

[1] Values are presented as least square means and SEM

[2] Number of samples: Control $n$ = 23. Probiotic $n$ = 18

[3] Treat = Treatment group, Segment = Intestinal segment, Treat x Segment = interaction between treatment and intestinal segment.

* Different lowercase letters indicate statistical significance ($p < 0.05$) between intestinal segments.

**Table S10.** Effect of early probiotic inoculation on hematology parameters in blood on day 23–24 and 35–36 [1,2].

| | Control | Probiotic | SEM | *P*-value[3] | | |
|---|---|---|---|---|---|---|
| | | | | Treat | Day | Treat x Day |
| Hematocrit (%) | | | | 0.41 | <0.001 | 0.30 |
| Day 23-24 | 35.6 | 33.8 | 1.0 | | | |
| Day 35-36 | 38.1 | 38.1 | 1.0 | | | |
| Red blood cells ($10^{12}$ cells/L) | | | | 0.13 | <0.001 | 0.92 |
| Day 23-24 | 5.9 | 5.6 | 0.2 | | | |
| Day 35-36 | 7.0 | 6.6 | 0.2 | | | |
| Hemoglobin (g/L) | | | | 0.27 | <0.001 | 0.36 |
| Day 23-24 | 106 | 100 | 2.9 | | | |
| Day 35-36 | 117 | 115 | 3.4 | | | |
| White blood cells ($10^9$ cells/L) | | | | 0.68 | <0.001 | 0.11 |
| Day 23-24 | 9.4 | 11.0 | 0.9 | | | |
| Day 35-36 | 16.0 | 14.8 | 1.5 | | | |
| Lymphocytes (%) | | | | 0.88 | 0.21 | 0.95 |
| Day 23-24 | 60.3 | 59.5 | 2.4 | | | |
| Day 35-36 | 57.4 | 57.0 | 2.3 | | | |
| Neutrophils (%) | | | | 0.90 | 0.21 | 0.71 |
| Day 23-24 | 35.0 | 34.2 | 2.1 | | | |
| Day 35-36 | 36.6 | 37.2 | 2.2 | | | |
| Monocytes (%) | | | | 0.29 | 0.17 | 0.17 |

| | | | | | | |
|---|---|---|---|---|---|---|
| Day 23-24 | 3.4 | 4.6 | 0.4 | | | |
| Day 35-36 | 4.5 | 4.5 | 0.4 | | | |
| Eosins (%) | | | | 0.80 | 0.002 | 0.57 |
| Day 23-24 | 0.73 | 0.66 | 0.1 | | | |
| Day 35-36 | 0.96 | 0.97 | 0.1 | | | |
| Neutrophil/Lymphocyte ratio (%) | | | | 0.66 | 0.32 | 0.73 |
| Day 23-24 | 60.9 | 63.1 | 7.1 | | | |
| Day 35-36 | 64.7 | 71.2 | 7.9 | | | |
| Reticulocytes ($10^9$ cells/L) | | | | 0.16 | <0.001 | 0.02 |
| Day 23-24 | 250 | 236 | 42 | | | |
| Day 35-36 | 40[a] | 78[b] | 11 | | | |
| Platelets ($10^9$ cells/L) | | | | 0.95 | 0.003 | 0.53 |
| Day 23-24 | 607 | 584 | 95 | | | |
| Day 35-36 | 374 | 426 | 67 | | | |

[1] Values are presented as least square means and SEM

[2] Number of samples: Control d23–24 = 23, d35–36 = 23. Probiotic d23–24 = 20, d35–36 = 18.

[3] Treat = Treatment group, Treat x Day = Interaction between treatment and day

Different lowercase letters indicate statistical significance ($p < 0.05$).
